# Integrating transcriptomics and metabolomics to reveal the protective effect and mechanism of Bushen Kangshuai Granules on the elderly people

**DOI:** 10.3389/fphar.2024.1361284

**Published:** 2024-07-29

**Authors:** Jun Hu, Fengmin Yang, Guang Yang, Juhua Pan, Yumeng Tan, Yalin Tang, Yongmei Liu, Hong Zhang, Jie Wang

**Affiliations:** ^1^ Department of Cardiology, Guang’anmen Hospital, China Academy of Chinese Medical Sciences, Beijing, China; ^2^ National Laboratory for Molecular Sciences, Center for Molecular Sciences, State Key Laboratory for Structural Chemistry of Unstable and Stable Species, Institute of Chemistry, Chinese Academy of Sciences, Beijing, China; ^3^ Research and Development Center of Traditional Chinese Medicine, Guang’anmen Hospital, China Academy of Chinese Medical Sciences, Beijing, China; ^4^ Institute of Basic Research in Clinical Medicine, China Academy of Chinese Medical Sciences, Beijing, China

**Keywords:** aging, Bushen Kangshuai Granules (BKG), traditional Chinese medicine, transcriptomics, metabolomics

## Abstract

**Background:** Aging is characterized by a decline in the adaptability and resistance of the body. In this study, Bushen Kangshuai Granules (BKG), as a kind of Chinese herbal formula, was developed and shown to alleviate aging-related symptoms.

**Methods:** Self-controlled study combined with RNA-seq and metabonomics were used to expound the efficacy and safety of BKG and revealed the regulation mechanism of BKG treating aging. *In vitro* experiments were used to confirm the analytical results. The aging cell model of AC16 cells were treated with D-galactose. The RT-qPCR was used to detect the impact of BKG on telomere length. The DCFH-DA staining was used for detecting intracellular ROS. The targeted signaling pathway was selected and verified using Western blot.

**Results:** After 8 weeks of treatment, BKG significantly reduced SOD level (*p =* 0.046), TCM aging symptoms (*p <* 0.001) and TNF-α level (*p =* 0.044) in the elderly participants. High-throughput sequencing showed that BKG reversed the expression of 70 and 79 age-related genes and metabolites, respectively. Further enrichment analysis indicated that BKG downregulated the *PI3K*-*AKT* signaling pathway, extracellular matrix (ECM)-receptor interaction, and Rap1 signaling pathway, while up-regulating sphingolipid metabolism. The results of *in vitro* experiments show that, after D-gal treatment, the viability and telomere length of AC16 cells significantly decreased (*p <* 0.05), while the expression of ROS increased (*p <* 0.05), BKG significantly increased the telomere length of AC16 cells and reduced the level of ROS expression (*p <* 0.05). In addition, BKG decreased the expression of THBS1, PDGFRA, and EPS8L1(*p <* 0.05), consistent with the RNA-seq results. Our results also showed that BKG affects *PI3K*-*AKT* signaling pathway.

**Conclusion:** BKG can significantly improve aging-related symptoms and increase SOD levels, which may be associated with the reversal of the expression of various aging-related genes. The *PI3K*-*AKT* signaling pathway and sphingolipid metabolism may be potential mechanisms underlying BKG anti-aging effects.

## Background

Aging is a progressive degenerative change in the physiological structure and functional decline of the body that occurs with increasing age. It is a process of reduced adaptability and decreased body resistance (Raz et al., 2010; Dodig et al., 2019). Most age-related diseases associated with the aging of the body or cellular senescence include atherosclerosis, osteoarthritis, cancer, Alzheimer’s disease, and chronic obstructive pulmonary disease (Childs et al., 2017). With the development of modern medical technology, research on aging mechanisms has advanced, shifting from theories focused on DNA damage, telomere shortening, and stem cell damage in earlier years, to various levels such as immune and vascular aging, dysbiosis, impaired autophagy, chronic inflammation, and non-coding RNA (Dodig et al., 2019; Zhang et al., 2021; Baechle et al., 2023). Several therapeutic anti-aging strategies have been developed, including anti-inflammatories, antioxidants, autophagy, telomerase, and mitochondrial activators, as well as stem cell, microbial, and noncoding RNA therapies (Mishra et al., 2022; Rosen and Yarmush, 2023). However, most of these therapies are still being explored and lack relevant clinical evidence. Further in-depth research and verification are required. Additionally, many monotherapy clinical trials have failed to influence disease progression or symptoms. Therefore, the complex pathophysiology of aging may require a combination therapy rather than a single treatment.

Traditional Chinese Medicine (TCM) has a history of more than two thousand years in China and has accumulated a wealth of experience in promoting health and longevity and delaying aging. Chinese herbal medicines have the advantage of affecting multiple components and targets. Their effects are often not limited to a single disease or pathological process. TCM improvements are generally comprehensive, making it more suitable for improving multiple symptoms caused by physiological decline. In traditional Chinese medical theory, kidneys are closely related to growth, development, and reproduction. It is believed that an abundance of kidney Qi leads to longevity, whereas a deficiency of kidney Qi leads to premature aging. Various symptoms of aging and the resulting diseases are considered to be mainly caused by “kidney deficiency.” Therefore, TCM often adopts a “tonifying the kidney” approach to delay aging. Modern research suggests that the essence of the kidney in TCM not only includes the urinary system, but also incorporates some functions of the “hypothalamic-pituitary-gonadal” endocrine axis. Currently, various kidney-tonifying herbal formulae and their active ingredients in Chinese medicine have shown encouraging anti-aging effects. For example, the Ba Zhi Bu Shen formula can alleviate epigenetic aging and prolong a healthy lifespan in naturally aging mice (Mao et al., 2023). Ba Zi Bu Shen capsules alleviate cognitive impairment by inhibiting microglial activation and cellular senescence (Mao et al., 2023). Bu Shen Yi Zhi Fang alleviates cognitive impairment in Senescence Accelerated Mouse-Prone 8 (SAMP8) mice and regulates inflammation, oxidative stress, and neuronal apoptosis. Bu Shen Yi Zhi Fang improved mitochondrial dysfunction and oxidative stress in D-galactose-induced aging rats via the AMPK/Sirt1 signaling pathway (Hou et al., 2018; Liao et al., 2023). Therefore, kidney-tonifying herbal formulae have the potential to treat aging.

Bushen Kangshuai Granules (BKG) comes from the traditional Chinese medicine prescription “Yougui Pill,” which comes from the *Complete Compendium of Zhang Jingyue*. Studies have shown that Yougui Pill has clinical significance for a variety of age-related diseases. For example, Yougui pill can inhibit cartilage degeneration by enhancing the activation of TGF-β/Smad signaling pathway, and is expected to be a good choice for the treatment of osteoarthritis (Zhang et al., 2017). Yougui pill treatment can activate the expression of VEGF and β-catenin to promote bone formation, increase the load-bearing capacity of the femoral head, and effectively inhibit the growth and differentiation of osteoclasts (Zhang P. et al., 2019). In addition, Yougui Pill can also improve glucocorticoid-induced hypothalamic-pituitary-adrenal axis inhibition and reduce the apoptosis of anterior pituitary cells, the mechanism of which is related to the regulation of mitochondria-mediated apoptosis pathway (Ji et al., 2016). BKG is composed of kidney-tonifying botanical drugs such as Panax ginseng, Cordyceps sinensis, pilose antler glue, goji berries, and Cistanche deserticola. Research has shown that some botanical drugs and active ingredients of BKG exert varying degrees of therapeutic effects on age-related neurogenesis and reproductive decline. For example, Panax ginseng can regulate hormone metabolism, improve age-related cognitive impairment, and prevent premature ovarian failure (Shi et al., 2012; Zhu et al., 2015). Cordyceps sinensis possesses antioxidant and anti-aging activities, and can prolong fruit flies lifespan, improve sexual function, and enhance learning and memory in mice (Ji et al., 2009; Zhu et al., 2020). Pilose antler glue is a traditional tonic food or medicine used in East Asia for the treatment of age-related diseases. The methanol extract of pilose antler glue improves Parkinson’s disease by inhibiting oxidative stress and neuroinflammation (Liu et al., 2021). Goji berry (Lycium barbarum) treatment improves brain plasticity in aging rats and mitigates macular degeneration in aging mice (Ruíz-Salinas et al., 2020). Cistanche deserticola restores gut-brain axis and alleviates cognitive decline in an aging mouse model, while preventing osteoporosis (Gao et al., 2021; Wang et al., 2021).

**FIGURE 1:**
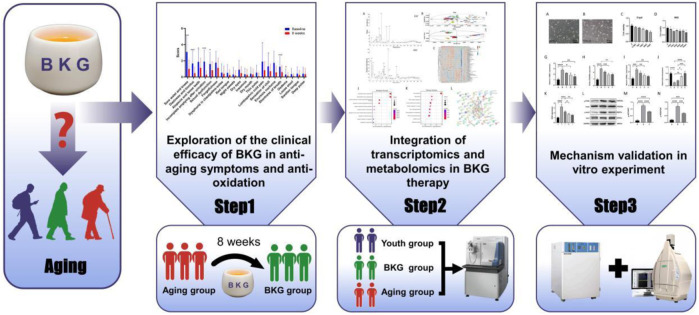
The flow chart of study.

In traditional medical practices, TCM places greater emphasis on improving age-related symptoms. Therefore, to evaluate BKG effect on improving multiple symptoms associated with aging, we first conducted a self-controlled study to evaluate the efficacy and safety of BKG in treating aging-related symptoms. We referred to the “Self-Rating Scale for Aging” and developed a scoring scale (see appendix) that includes 21 typical aging-related symptoms such as soreness and weakness of the waist and knees, dizziness and tinnitus, hair loss, loose teeth, and residual urine after urination. We evaluated BKG efficacy by assessing the degree of improvement in these symptoms through interventions in an elderly population. Additionally, we observed BKG effects on objective indicators of aging, such as Superoxide Dismutase (SOD), Tumor Necrosis Factor-alpha (TNF-α), sex hormones, blood routine, liver and kidney function, to evaluate BKG safety. In addition, we conducted RNA-seq, metabolomics studies, and *in vitro* experiments to further analyze the mechanism and potential targets of BKG therapy for aging (Figure 1).

## Materials and methods

### Participants recruitment

Thirty elderly individuals were recruited from the community through advertising between December 2019 and December 2020. The inclusion criteria were as follows: 1) 40–80 years, and 2) meet diagnostic criteria for aging and kidney deficiency syndrome (Table 1). The diagnostic criteria for kidney deficiency syndrome were referenced to the “Guidelines for Clinical Research of New Traditional Chinese Medicines” (2002 edition). This study was approved by the Ethics Committee of Guang’anmen Hospital, China Academy of Chinese Medical Sciences (2019-224-KY). The study was conducted in accordance with the National Research Committee and the 1964 Declaration of Helsinki and its subsequent amendments or comparable ethical standards. All volunteers provided informed consent before participating in the study.

**TABLE 1:** Diagnostic criteria for kidney deficiency syndrome.

Kidney deficiency	Items
Primary Symptoms	Symptoms include soreness and weakness in the waist and knees, frequent nocturia, decreased sexual function and energy
Secondary Symptoms	Symptoms include dizziness, tinnitus, hair loss or graying, loose teeth, residual urine or urinary incontinence, cold and painful lower back and abdomen, cold limbs, forgetfulness, pale tongue, and weak pulse

Exclusion criteria included: 1) Individuals with poorly controlled hypertension (systolic blood pressure ≥160 mmHg or diastolic blood pressure ≥100 mmHg); 2) Patients with concomitant cardiovascular, cerebrovascular, hepatic, renal, endocrine, or hematological disorders, as well as those with abnormal liver function (Alanine or Aspartate aminotransferase (ALT or AST) values > 1.5 times the upper limit of normal) or renal dysfunction; 3) Patients with mental disorders, depression, or anxiety disorders; 4) Pregnant or lactating women; 5) Patients who have undergone surgery within the previous month; 6) Patients with bleeding tendencies, disseminated intravascular coagulation (DIC), abnormal NR values, or thrombocytopenia; 7) Patients who have participated in other clinical trials within the previous month; 8) Patients with allergies or hypersensitivity to the components of the test drug.

### Intervention

In this study, we use chinese medicine formula granules to control the quality of Chinese medicine. BKG formula granules were purchased from Sichuan Neo-Green Pharmaceutical Technology Development Co., Ltd. All Chinese medicines were of controlled quality, and test reports were provided, including thin-layer identification results and active ingredient content (Additional file3). BKG is composed of Chinese herbal medicines such asgelatinum Cervi Cornu (10 g), Lycium barbarum (20 g), Herba Cistanches (20 g), Morinda officinalis (10 g), Paeonia lactiflora (20 g), and Citrus reticulata peel (10 g). The formula was prepared into granules according to the above prescription by the granule pharmacy of guang’anmen Hospital, China Academy of Chinese Medical Sciences. The medicine was dissolved in hot water and administered twice daily (one sachet each time) for 8 weeks. During the trial period, the use of any other form of health products was prohibited.

### Sample size estimation

Serum SOD level is often used as one of the indicators for evaluating the effectiveness of anti-aging drugs. Data from the literature shows that the serum SOD level in elderly patients is 81.83 U/mL, with a standard deviation of 14.8 U/mL. It is expected that after treatment with kidney-replenishing and anti-aging herbal medicine, the SOD level will increase to 92.43 U/mL. Assuming a Type I error (α) of 0.05, a Type II error (β) of 0.1, and using the formula n = 2σ×f (α,β)/(μ1-μ2)2, where σ = 14.8, μ1 = 81.83, μ2 = 92.43, at α = 0.05, β = 0.1, f (α,β) = 10.5, and considering a dropout rate of 20%, the final calculated sample size is 26 cases, and a comprehensive analysis indicates that including 30 subjects is reasonably acceptable.

### Outcome measures

The primary outcome measure is serum SOD level. Other outcome measures included the following: 1) TCM aging symptom score. The aging symptom scale of the TCM was used, which included 21 symptom items each with an evaluation score of 0, 2, 4, or 6 (see Additional file 1: Supplementary Table S1). 2) Physical fitness and fatigue index. The Borg Scale was used to assess physical fitness and fatigue levels of the participants. 3) TNF-α levels. 4) Hormone levels. 5) Complement levels. 6) Safety indicators including routine blood examination, liver function [ALT, AST, and alkaline phosphatase (ALP)], and kidney function (blood urea nitrogen (BUN), Creatinine (Cr), and Uric Acid (UA).

### RNA-seq approach

#### Sample collections

To explore the transcriptomic differences between young individuals, elderly individuals and BKG therapy, 20 volunteers were recruited from Guang’anmen Hospital, China Academy of Chinese Medical Sciences. Among them, 10 volunteers aged 23–29 years served as the youth group (Group A). Another group of 10 volunteers aged 60–71 years served as the aging group (Group B). The volunteers in Group B received administration of BKG (100 mL per dose) twice a day for a continuous duration of 8 weeks, forming the BKG group (Group C). In the early morning, peripheral blood samples were collected from volunteers in vacuum tubes containing Ethylenediaminetetraacetic acid (EDTA) anticoagulant. The peripheral blood mononuclear cells were then extracted using the TRIzol method (15596018, Invitrogen, Carlsbad, CA, United States) and immediately stored at −80°C.

#### RNA extraction, library construction and sequencing

Total RNA was extracted using a TRIzol reagent kit (Invitrogen) according to the manufacturer’s protocol. RNA quality was assessed using an Agilent 2100 Bioanalyzer (Agilent Technologies, Palo Alto, CA, United States) and verified by RNase-free agarose gel electrophoresis. After total RNA was extracted, eukaryotic mRNA was enriched using oligo (dT)beads, whereas prokaryotic mRNA was enriched by removing rRNA using the Ribo-ZeroTM Magnetic Kit (Epicenter, Madison, WI, United States). The enriched mRNA was then fragmented into short fragments using a fragmentation buffer and reverse transcribed into cDNA using random primers. Second-strand cDNA was synthesized using DNA polymerase I, RNase H, dNTP, and a buffer. The cDNA fragments were purified using the QiaQuick PCR extraction kit (Qiagen, Venlo, Netherlands), end-repaired, poly(A) added, and ligated to Illumina sequencing adapters. The ligation products were size-selected by agarose gel electrophoresis, polymerase chain reaction (PCR)-amplified, and sequenced using Illumina HiSeq2500 (Gene *Denovo* Biotechnology Co., Guangzhou, China).

#### Bioinformatics analysis

Reads obtained from sequencing machines included raw reads containing adapters or low-quality bases that affected subsequent assembly and analysis. To obtain high-quality clean reads, they were filtered using fastp (Chen et al., 2018) (version 0.18.0). An index of the reference genome was built and paired-end clean reads were mapped to the reference genome using HISAT2.2.4 (Kim et al., 2015) with “-rna-strandness RF” and other parameters set as a default. The mapped reads for each sample were assembled using StringTie v1.3.1 (Pertea et al., 2015; Pertea et al., 2016) in a reference-based approach. For each transcription region, the fragment per kilobase of transcript per million mapped reads (FPKM) value was calculated to quantify its expression abundance and variations using StringTie software. RNAs differential expression analysis was performed using the DESeq2 (Love et al., 2014) software between two different groups and edgeR (Robinson et al., 2010) between the two samples. The genes/transcripts with the parameter of false discovery rate (FDR) > 0.05 and absolute fold change ≥2 were considered differentially expressed genes. Gene Ontology (GO) database and Kyoto Encyclopedia of Genes and Genomes (KEGG) were used for biological process and pathway enrichment analyses, respectively. The calculated *p*-value went through FDR Correction, taking FDR ≤0.05 as a threshold. Pathways and GO terms that met this criterion were defined as significantly enriched differentially expressed genes (DEGs).

### Metabolomic methods

#### Human subject and preparation of clinical samples

To explore the Metabolomic differences between young individuals, elderly individuals and BKG therapy, fifty six volunteers were recruited from Guang’anmen Hospital, China Academy of Chinese Medical Sciences. Among them, 30 volunteers aged 23–29 years served as the youth group (Group A). Another group of 30 volunteers aged 42–71 years served as the elderly group (Group B). Volunteers from Group B who received treatment were referred to as the BKG group (Group C). Blood samples were collected after 12 h of fasting in vacuum tubes containing EDTA anticoagulant. The mixture was shaken slowly and allowed to stand at room temperature for 1 h. The plasma supernatant samples were collected after centrifuging at 1,000 g for 10 min at 4°C and stored immediately at −80°Cuntil analysis.

#### Pretreatment of plasma samples

The plasma samples were thawed at 4°C. 200 μL of plasma sample was mixed with 600 μL of acetonitrile for protein precipitation. The mixture was vortexed for 2 min and sonicated on ice for 10 min. Then mixture was allowed to stand for 2 h at −20°C and then centrifuged at 13,000 rpm for 10 min at 4°C. 150 μL supernatant was added into the injection bottle for testing. Simultaneously, an equal amount of supernatant was mixed with a quality control (QC) sample for testing.

#### Liquid chromatography–mass spectrometry analysis

Plasma samples were analyzed using an Orbitrap Fusion Lumos Tribrid Mass Spectrometer (Thermo Fisher Scientific, City, Country). Waters ACQUITY UPLCBEH C18 chromatographic column (2.1 × 100 mm, 1.7 µm) was used to separate with 0.3 mL/min. Mobile phases A and B were 20 mM aqueous ammonium acetate solution and acetonitrile containing 0.1% formic acid, respectively. The gradient elution program was set as follows: 0–20 min, 95%–70% B; 20–21 min, 70%–50% B; 21–24 min, 50% B; 24–25 min, 50%–95%; 25–30 min, 95% B. The injection volume is 5 μL and the column temperature was set at 25°C. Mass spectrometry detection with an Orbitrap detector used an electrospray ionization source (ESI) in positive and negative ionization modes (ESI+, ESI-). The auxiliary spray ionization desolvation gas was N_2_. Steam, auxiliary, and sweep gases flow rates were 35 Arb, 10Arb and 2Arb, respectively. Vaporizer and ion transfer tube temperatures were set 275°C and 325°C, respectively. Positive and negative ion modes spray voltages were 3200 V and −2500 V, respectively. Mass spectrometry acquisition range m/z was set 60–1,000 Da. The QC samples were tested once every ten samples.

#### Data pre-processing and multivariate analysis

Pretreatment of raw data was performed using Compound Discoverer TM 3.1(CD 3.1), including peak discovery, matching, and identification. Peaks with absolute intensities>105 were identified and preserved. Finally, a list of peaks containing the compound name, formula, molecular weight, and intensity was obtained. SIMCA-P software (version 14.0, Umetrics) was used for multivariate analysis. Differential metabolites were screened by principal component analysis (PCA) and orthogonal projection to latent structures-discriminant analysis (OPLS-DA) (Yang et al., 2021; Kui et al., 2022). The OPLS-DA model was further evaluated using permutation testing (n = 200).

### Differential metabolite analysis

The value of the projected variables (VIP) obtained from OPLS-DA was used to screen differential metabolites and VI*P>*1 was one of the screening criteria. The other screening criterion was a *p*-value<0.05, which indicated that the two sets of data were statistically significant. The *p*-value<0.05 were processed using the Mann-Whitney U-test (SPSS 25.0). Compounds with VI*P>*1 and *p <* 0.05 were considered differential metabolites. Hierarchical cluster analysis (HCA) was conducted to identify the differential metabolites and their changing trends. Online tools such as The Human Metabolome Database (HMDB) and MetaboAnalyst 5.0, were used to analyze metabolic pathways.

### Cells and reagents

AC16 cells (STCC13101P), DMEM cell culture medium (G4511), FBS(G8001), D-(+)-Galactose (GC205005), Cell Counting Kit-8(CCK8, G4103), SYBR Prime qPCR set (G3326), Super ECL plus Western blotting kit (G2074), TRIzol (G3013), RIPA lysis buffer (G2002), 0.25% trypsin digestion solution (G4013), SDS-PAGE kit (G2043), Annexin V-FITC/PI apoptosis detection kit (G1511) were purchased from Servicebio (Wuhan, China). Cell culture dishes were purchased from Corning (United States). Primers used in qPCR were synthesized from Servicebio (Wuhan, China).

### Cell treatments

AC16 cells were cultured in DMEM cell culture medium with 10% FBS in 5% CO_2_ and 37°C cell cultural incubator. About 2×10^6^ AC16 cells were separated into 6 cm dishes for 24 h before D-gal addition. Then, BKG was added into the medium with different concentrations, and PBS was used as control. Twenty-four hours later, RNA and protein was extracted respectively.

### CCK8 experiment

CCK8 experiment was performed according to the protocol of manufacture. In brief, about 105 cells per well were seeded into the 96 cells culture plate respectively. Different concentration of D-gal and BKG were added 24 h later. CCK8 solution was added at the 24 h time point after D-gal and BKG intervention. OD.450 was detected using microplate reader. There were five replicates for each treatment.

### Real-time PCR

RNA was extracted by RNA lysate at 24 h after D-gal and BKG exposure. Real-time PCR was performed with the following condition: 95°C for 30 s; 40 cycles of 95°C for 15 s, 60°C for 30 s. Melting curve analysis was performed with the following condition: 95°C for 1 min, 60°C for 1 min, increasing from 60°C to 95°C (+0.5°C and for 5 s per cycle). Method of 2^(-ΔΔCt)^ was used to quantify the relative expression levels of genes. GAPDH was used as the internal reference. Primers list were listed as in Supplementary Table S5.

### Western blotting

AC16 cells were exposed to 0, 50 and 100 μM D-galactose respectively for 24 h. Protein was extracted using RIPA lysis buffer. After BCA protein quantification, equal amount of protein samples was loaded for SDS-PAGE. Then, protein in the gel was wet-transferred to 0.22 μm PVDF membrane. After blocking with 5% non-fat milk within TBST for 1 h at RT, the membrane was incubated with first antibody at 4°C overnight. Next, the membrane was thoroughly washed with TBST and incubated with second antibody for 1 h at RT. Finally, Fusion FX5 Spectra (Vilber, France) system was used to capture pictures developed by ECL solution. ImageJ was used to quantify the grey scale of images.

### Statistical analysis

SPSS26.0 software was used for statistical analysis, and adverse reactions were analyzed among participants who withdrew owing to adverse events. The measurement data were statistically described by means of standard deviation, and the counting data were statistically described by frequency or percentage. The paired sample t-test was conducted for the results before and after the experiment, and the independent sample t-test was used for comparisons between groups. The statistical tests will be double-sided, and *p <* 0.05 will be considered statistically significant. Frequency was used to describe the counting data, frequency was used to describe the counting data by Chi-square test, and Fisher exact probability method was used.

## Results

### BKG attenuated age-related symptoms

**FIGURE 2:**
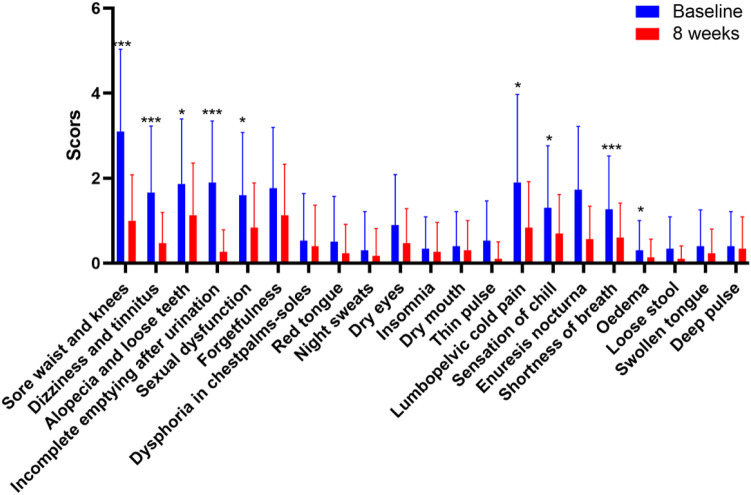
Improvement in the ageing-related symptoms after treatment.*p < 0.05, **p < 0.01, ***p < 0.001; *n* = 30.

The study included 30 participants who demonstrated good compliance and completed the experiment. Ultimately, all 30 participants successfully completed the study without any dropouts. Among the 30 participants, 11 were males and 19 were females, aged 42–71. The average age of the participants was 51.8 ± 6.68 years. We evaluated improvements in age-related symptoms using a self-designed aging symptom scale in TCM (Supplementary Table S1). As shown in Table 2, TCM aging symptom scores were 22.93 ± 11.36 and 10.27 ± 7.00 before and after treatment, respectively, with a statistically significant difference (*p <* 0.001) between the two groups, indicating a notable improvement in aging-related symptoms after treatment. Specifically, significant improvements (*p <* 0.05) were observed in symptoms including sore waist and knees, dizziness, alopecia, dysuria, residual loss of function, abdominal pain, chills, dyspnea, and edema (Figure 2). Additionally, after treatment, the Borg fatigue index scores of the participants decreased significantly from 1.97 ± 1.29 to 0.42 ± 0.49, with a statistically significant difference (*p <* 0.001). This indicated a significant improvement in the fatigue levels of the participants.

**TABLE 2:** Improvement of efficacy and safety indicators.

	Baseline	8 Weeks	*p*-value
Aging symptom score of TCM	22.93 ± 11.36	10.27 ± 7.00	<0.001
Brog fatigue index score	1.97 ± 1.29	0.42 ± 0.49	<0.001
Superoxide dismutase (SOD, U/mL)	199.33 ± 11.78	203.9 ± 14.38	0.046
Interleukin-6 (IL-6, pg/mL)	3.18 ± 0.94	2.84 ± 1.78	0.376
Tumor necrosis factor-alpha (TNF-α, pg/mL)	6.92 ± 1.87	6.32 ± 1.4	0.044
Testosterone (ng/mL)	2.11 ± 2.6	2.37 ± 2.99	0.089
Estradiol (pg/mL)	40.1 ± 44.26	59.37 ± 80.85	0.149
Complement component 3 (C3, g/mL)	0.92 ± 0.17	0.91 ± 0.19	0.669
Complement component 4 (C4, g/mL)	0.26 ± 0.21	0.25 ± 0.15	0.893
Alkaline phosphatase (ALP, U/L)	82.97 ± 22.5	78.83 ± 20.87	0.071
Aspartate transaminase (AST, U/L)	24.22 ± 8.5	21.26 ± 7.07	0.039
Alanine transaminase (ALT, U/L)	24.72 ± 14.76	22.97 ± 11.33	0.309
Creatinine (Cr, imol/L)	63.7 ± 11.49	66.57 ± 13.53	0.003
Blood urea nitrogen (BUN, mmol/L)	5.11 ± 1.07	5.14 ± 1.08	0.893
Red blood cells (RBC, × 10^12^/L)	4.87 ± 0.47	4.79 ± 0.45	0.152
White blood cell count (WBC, × 10^9^/L)	5.7 ± 1.1	5.56 ± 1.32	0.393
Hemoglobin (g/L)	144.13 ± 12.73	145.03 ± 13.85	0.506
Platelets (PLT, × 10^9^/L)	246.97 ± 51.68	248.57 ± 52.21	0.735
Lymphocyte percentage (%)	32.02 ± 6.16	31.78 ± 5.05	0.797
Albumin (ALB, g/L)	45.84 ± 1.69	44.63 ± 2.17	0.009
glucose (GLU, mmol/L)	5.59 ± 0.57	5.7 ± 0.74	0.191

### BKG improved antioxidant function

In terms of antioxidant function, SOD levels of the participants increased significantly after treatment (*p =* 0.046). The IL-6 level decreased after treatment, but the difference was not statistically significant (*p =* 0.376), while the TNF-α level significantly decreased after treatment (*p =* 0.044). In terms of sex hormones, testosterone and estradiol levels showed an increasing trend after treatment; however, this was not statistically significant (*p =* 0.089 and *p =* 0.149, respectively). More specifically, after treatment, testosterone levels showed an increasing trend in the 11 male patients, estradiol levels showed a decreasing trend, and the T/E2 ratio showed an increasing trend. After treatment, testosterone levels showed an increasing trend in the 19 female patients, estradiol levels showed an increasing trend, and the T/E2 ratio showed a decreasing trend. The levels of complements C3 and C4 after treatment were similar to those observed before treatment. No significant changes were observed in other indicators. No serious adverse events occurred in terms of safety, and the participants showed a significant decrease in AST levels after treatment (*p =* 0.039), suggesting that the treatment may have a certain hepatoprotective effect. The participants showed a statistically significant increase in Cr after treatment compared to that before treatment (*p =* 0.003), and BUN showed an increasing trend (*p =* 0.893), indicating that treatment may have a certain impact on kidney function. No significant changes were observed in other indicators.

### BKG treatment reversed the expression of aging-related metabolites

**FIGURE 3:**
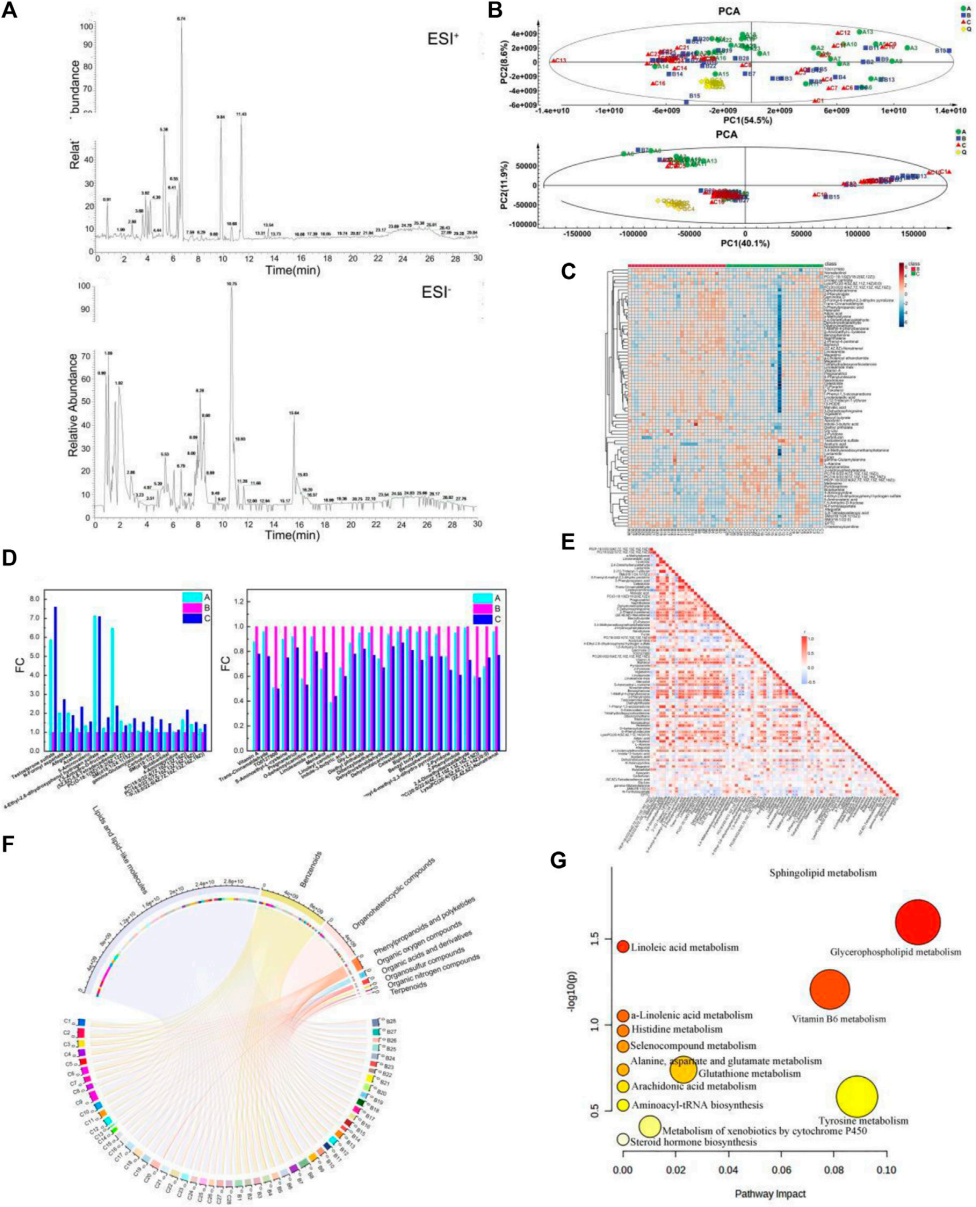
Metabolic signature in the youth group, elderly and BKG groups. **(A)** The base peak chromatograms of the plasma containing positive and negative ion modes. **(B)** The score scatter plot of PCA. **(C)** hierarchical cluster analysis between B and C groups. **(D)** Variation trend of different groups. **(E)** A heat map of the correlation between the differential metabolites. **(F)** Chord diagram of the Differential metabolites. **(G)** Metabolomics pathway analysis of differential metabolites in the B-C group (the nodes are colored based on the *p*-value, and the node size indicates impact values). **(A)** youth group. **(B)** elderly group. **(C)** BKG group.

The demographic and clinical characteristics of the volunteers enrolled in metabolomics and transcriptomic studies were listed in Table 3. Metabolites were analyzed by liquid chromatography–mass spectrometry (LC-MS) in four groups of plasma samples: groups A, B, C and D (youth, aging, BKG, and QC groups, respectively). The base peak chromatograms of the plasma containing positive and negative ion modes are shown in Figure 3A. QC samples were used to control the quality of the inspection system and were injected into 10 test samples. Principal component analysis (PCA) is an unsupervised data analysis method used to observe the overall distribution of samples and the stability of the entire analysis process. Therefore, PCA was used to analyze the overall data of groups A, B, C, and D. The aggregation of QC test results reflects the stability of the system throughout the sample testing process. In the score scatter plot of PCA, the QC samples were closely clustered under ESI+ and ESI- (Figure 3B), indicating good instrument detection stability during the experiment.

**TABLE 3:** Clinical characteristics of volunteers enrolled in metabolomics and transcriptomic studies.

Participants	Youth group	Elderly group	BKG group
RNA-seq			
sample size	10	10	10
Age	24.70 ± 1.49	52.60 ± 5.93	52.60 ± 5.93
Sex (male%)	6 (60.00%)	5 (50.00%)	5 (50.00%)
Hypertension (%)	0 (0.00%)	1 (10.00%)	1 (10.00%)
Hyperlipidemia (%)	0 (0.00%)	1 (10.00%)	1 (10.00%)
Diabetes (%)	0 (0.00%)	0 (0.00%)	0 (0.00%)
Metabolomics			
sample size	30	30	30
Age	25.07 ± 1.48	51.8 ± 6.68	51.8 ± 6.68
Sex (male%)	17 (56.67%)	11 (36.67%)	11 (36.67%)
Hypertension (%)	0 (0.00%)	3 (10.00%)	3 (10.00%)
Hyperlipidemia (%)	0 (0.00%)	5 (16.67%)	5 (16.67%)
Diabetes (%)	0 (0.00%)	0 (0.00%)	0 (0.00%)

Multivariate statistical analyses were performed using unsupervised PCA analysis, supervised partial least squares analysis (PLS-DA) and orthogonal partial least squares analysis (OPLS-DA). First, PCA was performed on Groups B and C to obtain an overview of the responses before and after BKG administration. Multivariate statistical analysis results under ESI + are shown in Supplementary Table S2 and Supplementary Figure S1. The PCA data were reduced to two principal components and there were two outliers. PCA failed to show a clear separation between groups B and C owing to the complexity of the clinical samples. Supervised OPLS-DA was conducted to analyze the data of groups B and C, and the two groups of samples were clearly separated. The model exhibited a certain predictive performance, with Q2 = 0.402. A permutation test was conducted to validate the OPLS-DA model. The intercept value of Q2 was less than 0 (−0.217), and the model was effective. Multivariate statistical analysis results under ESI- are shown in Supplementary Table S3 and Supplementary Figure S2. The PCA data were reduced to two principal components and there were four outliers. PCA failed to show a clear separation between groups B and C owing to the complexity of the clinical samples. Supervised OPLS-DA was conducted to analyze the data of groups B and C, and the two groups of samples were clearly separated. A permutation test was conducted to validate the OPLS-DA model. The intercept value of the Q2 was less than 0 (−0.325) and the model is effective.

A total of 9,186 and 2,721 material peaks were detected in the positive and negative modes, respectively. Finally, 1,843 and 394 metabolites were identified in positive and negative modes, respectively, as retrieved from the databases. The OPLS-DA model indicated that metabolites were primarily responsible for the separation. Therefore, VI*P>*1 of the OPLS-DA model was generated to screen for differential metabolites as the standard. The data were then screened for metabolites with statistical differences using the Mann-Whitney U test. Differential metabolites with VI*P>*1 and *p*-value<0.05 in positive mode were 70, and those with VI*P>*1 and *p*-value<0.05 in negative mode were nine. The Differential metabolites are listed in Supplementary Table S4.

Among the differential metabolites, 27 in group C were significantly upregulated and 52 in group C were significantly downregulated compared with those in group B. Upregulated metabolites included testosterone sulfate, N-Formylaspartate, Afegostat, Aceturic acid, 5-Aminovaleric acid, etc. Highly correlated differential metabolites were clustered together, and the relationship between the differential metabolites was visualized using hierarchical cluster analysis (HCA). Therefore, an HCA heat map was used to cluster the differential metabolites obtained from the above analyses. Although there was a partial overlap between groups B and C samples, HCA revealed differences in metabolite concentrations between the two groups (Figure 3C). Based on vertical metabolite clustering, two broad categories were in the first hierarchical cluster. The upregulated differential metabolites were clustered into one group, except for PC(O-18:1 (9Z)/18:2 (9Z,12Z)) and noradrenaline. Downregulated differential metabolites were clustered in other groups.

Compared with group B, 44 metabolites had a similar variation trend in groups C and A. Among them, concentrations of 18 metabolites were upregulated and that of 27 were downregulated. Upregulated metabolites included Testosterone sulfate, N-formyl aspartate, Afegostat, Aceturic, 5-Aminovaleric, 4-Ethyl-2,6-dihydroxyphenyl hydrogen sulfate (Figure 3D). Downregulated metabolites included Vitamin A, Trans-Cinnamaldehyde, TO0127900, S-Aminoethyl-L-cysteine. Compared to before administration, the serum metabolite concentrations of middle-aged and elderly participants after BKG administration were closer to those of the younger group.

Correlation analysis can be used to measure the degree of correlation between different metabolites. The Pearson correlation coefficient was used to measure the degree of linear correlation between the two metabolites and to understand the relationships between the metabolites during changes in biological states. A heat map of the correlation between the differential metabolites was plotted based on the Pearson correlation coefficient between the compounds (Figure 3E). The co-mediation relationship between various metabolites is visually revealed in a heat map of the correlation. The darker the red in the square, the stronger the positive correlation. The darker the blue color in the square, the stronger the negative correlation. The white squares indicate a weak correlation between the compounds. The heat map of the correlation presented both the Pearson correlation coefficients among the levels of metabolites and their structural similarities. For example, g-toferol, linolenolaidic acid, and 13-HODE are lipids and lipid-like molecules with strong positive correlation.

The Differential metabolites were classified and visualized using a chord diagram. All 79 metabolites were classified as lipids and lipid-like molecules, benzenoids, organoheterocyclic compounds, phenylpropanoids and polyketides, organic acids and derivatives, organic oxygen and organosulfur compounds, and terpenoids (Figure 3F). Among these, the proportions of lipids, lipid-like molecules, benzenoids, and organoheterocyclic compounds were relatively large. In addition, MetaboAnalyst, a commercial database, was used for pathway analysis. A bubble map was constructed to display significantly different metabolic pathways, including multiple metabolic pathways (Figure 3G), such as sphingolipid, glycerophospholipid, Vitamin B6, glutathione, and tyrosine metabolism. The specific results for the metabolic pathways are shown in Table 4.

**TABLE 4:** Metabolic pathways of Differential metabolites.

Pathway name	Match status	*p*	-log(*p*)	FDR	Impact	Trend
Sphingolipid metabolism	3-Dehydrosphingosine,SM(d18:1/22:0)	0.01	2.13	0.62	0.08	↑
Glycerophospholipid metabolism	PC(18:0/22:4 (7Z,10Z,13Z,16Z)) PC(16:0/22:5 (7Z,10Z,13Z,16Z,19Z)) PC(20:0/22:6 (4Z,7Z,10Z,13Z,16Z,19Z)) LysoPC(20:4 (5Z,8Z,11Z,14Z)/0:0)	0.02	1.68	0.88	0.11	↑ or ↓
Linoleic acid metabolism	PC(18:0/22:4 (7Z,10Z,13Z,16Z)) PC(16:0/22:5 (7Z,10Z,13Z,16Z,19Z)) PC(20:0/22:6 (4Z,7Z,10Z,13Z,16Z,19Z))	0.03	1.50	0.89	0.00	↑ or ↓
Vitamin B6 metabolism	Pyridoxamine	0.06	1.25	1.00	0.08	↓
alpha-Linolenic acid metabolism	PC(18:0/22:4 (7Z,10Z,13Z,16Z)) PC(16:0/22:5 (7Z,10Z,13Z,16Z,19Z)) PC(20:0/22:6 (4Z,7Z,10Z,13Z,16Z,19Z))	0.08	1.09	1.00	0.00	↑ or ↓
Histidine metabolism	N-Formyl aspartate	0.10	1.01	1.00	0.00	↑
Selenocompound metabolism	L-(+)-Alanine	0.12	0.91	1.00	0.00	↓
Alanine, aspartate and glutamate metabolism	L-(+)-Alanine	0.17	0.78	1.00	0.00	↓
Arachidonic acid metabolism	PC(18:0/22:4 (7Z,10Z,13Z,16Z)) PC(16:0/22:5 (7Z,10Z,13Z,16Z,19Z)) PC(20:0/22:6 (4Z,7Z,10Z,13Z,16Z,19Z))	0.21	0.68	1.00	0.00	↑ or ↓
Tyrosine metabolism	Noradrenaline	0.24	0.62	1.00	0.09	↓
Aminoacyl-tRNA biosynthesis	L-(+)-Alanine	0.27	0.57	1.00	0.00	↓

### BKG treatment reversed the expression of aging-related genes

**FIGURE 4:**
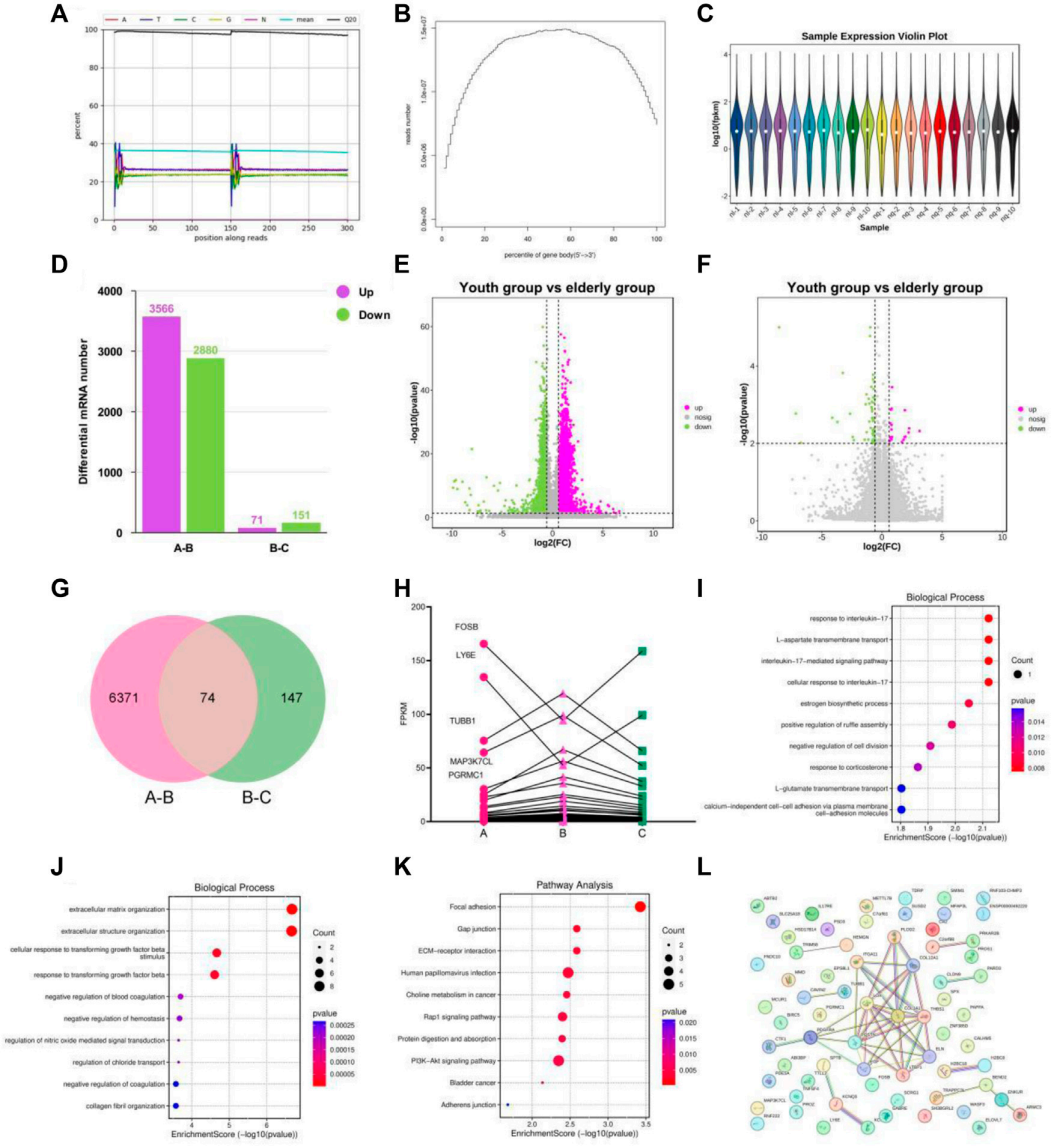
Transcriptional signature in the youth group, elderly and BKG groups. **(A**–**C)** Quality control on the raw reads. **(D)** Histogram of the numbers of differentially expressed genes (DEGs). **(E**–**F)** Volcano plot of DEGs between A-B groups and B-C groups. **(G)** Venn diagram of DEGs between 2 groups. **(H)** Variation trend of DEGs of A to C groups. **(I)** GO enrichment analyses of the upregulated aging genes affected by BKG. **(J)** GO enrichment analyses of the downregulated aging genes affected by BKG. **(K)** KEGG enrichment analyses of the upregulated aging genes affected by BKG. **(L)** Gene-gene interaction analysis on 70 genes. **(A)** youth group. **(B)** elderly group. **(C)** BKG group.

First, we performed quality control on the raw reads obtained from the sequencing run using FASTP software, filtering out low-quality data. After filtering, we observed that the content of four bases, A, T, G, and C, remained stable, indicating similar frequency of these four bases and stable sequencing process (Figure 4A). Gene randomness analysis showed that the reads were distributed relatively evenly across the different parts of the gene, indicating good randomness of mRNA fragmentation (Figure 4B). Based on the FPKM values of each gene, we present the expression distributions of different genes or transcripts in different samples using an expression distribution plot (Figure 4C). Next, we performed differential gene expression analysis using DESeq2 software and selected DEGs based on the criteria of FDR<0.05, and *p*-value > 1.5. Between groups A and B, 6,446 DEGs were significantly differentially expressed, with 3,566 upregulated and 2880 downregulated in aging-related genes (Figures 4D, E). Between groups B and C, 221 DEGs were significantly differentially expressed, with 71 genes upregulated and 151 DEGs downregulated in the BKG (Figure 4F). The gene expression levels are shown in Supplementary Tables S6, S7. We performed Venn analysis on these two gene sets and the results showed 74 overlapping genes (Figures 4G, 1–4). Among these 74 DEGs, 70 exhibited opposite expression trends between the A–B and B–C groups, suggesting that they may be potential targets of BKG treatment for aging. Based on the expression levels of these genes, we plotted a line graph and found that 50 DEGs exhibited a clear “high-low-high” trend in the young control, aging, and treatment groups, while 20 DEGs exhibited a “low-high-low” trend (Figure 4H; Table 5). This indicated that some dysregulated aging genes were corrected after BKG treatment.

**TABLE 5:** Genes which showed opposite expression trends between the A-B and B-C groups.

Gene symbol	logFC(A-B)	logFC(B-C)	Gene symbol	logFC(A-B)	logFC(B-C)
POSTN	6.006746832	−6.743712427	ELOVL7	1.100151027	−0.764058502
ELN	6.06608919	−5.845490051	PRKAR2B	1.077898918	−0.767915892
RNF103-CHMP3	−7.159871337	2.992466327	SPX	1.065368171	−0.758798102
ABI3BP	3.820178962	−4.129283017	FNDC10	−1.200178145	0.665740317
AC010323.1	−4.216317907	2.729876169	ZNF385D	0.792767386	−0.926274506
COL12A1	3.849665727	−1.992544195	CALHM5	0.91753784	−0.786293307
AP001273.2	−2.573185333	2.335934953	MFAP3L	1.112043151	−0.616090448
PROZ	−2.906890596	2	SH3BGRL2	0.86667911	−0.788240712
GABRE	2.652076697	−2.169925001	BIRC5	−0.835724721	0.785464112
METTL7B	−2.584962501	2.153805336	PARD3	0.963605763	−0.670306515
COL1A1	2.649092838	−1.699382411	PGRMC1	0.89743177	−0.714341617
ITGA11	1.610053482	−1.736965594	ABTB2	−0.785234803	0.797719625
CTF1	−1.37529132	1.898120386	H2BC18	0.860809223	−0.709888392
SLC25A18	−1.199937571	1.655351829	TNFSF4	1.011925514	−0.601975611
PAPPA	1.736965594	−1.133266531	GGTA1	0.973972694	−0.624292476
SCRG1	1.921997488	−0.915607813	MAP3K7CL	0.621755393	−0.969711835
TTLL2	1.415037499	−1.199769512	FOSB	−0.810246534	0.735710169
KCNQ3	1.490325627	−1.133266531	H2BC8	0.596324717	−0.992809622
IL17RE	−1.55884966	0.991810602	LTBP1	0.860158101	−0.686432484
LOX	1.428404723	−1.054076673	TRIM58	0.936757566	−0.629830618
BEND2	1.372932133	−1.048802466	THBS1	0.835307594	−0.704247626
HEMGN	1.928355927	−0.707345676	SUSD2	−0.629567803	0.908501646
HSD17B14	−1.256692879	1.067976794	CA2	0.847653979	−0.648441212
PSD3	1.96941168	−0.65702365	TUBB1	0.664298998	−0.822784075
TRAPPC3L	1.519234064	−0.793898063	ARMC3	0.757105065	−0.69086918
SMIM1	−1.213244039	0.989507626	SPTB	0.684422737	−0.737547209
CAVIN2	1.424673106	−0.822994778	MMD	0.645255376	−0.74251102
PROS1	1.427921031	−0.801416539	CLDN9	−0.753874729	0.628183029
PLOD2	1.442469627	−0.785771772	WASF3	0.681223042	−0.673249033
LY6E	−1.354736008	0.811594491	C7orf61	−0.762036755	0.596595162
RNF222	−1.055282436	0.980100443	TDRP	0.719821303	−0.614929784
EPS8L1	−1.172676662	0.79403578	EGF	0.712468511	−0.600743896
KCND3	1.210566986	−0.763932642	MCUR1	0.634806547	−0.64356785
PDGFRA	1.025535092	−0.899933045	CCDC200	1.065588342	0.789293751
C2orf88	1.21059525	−0.758293408	ANGPT2	1.403355694	0.765534746
PDE5A	1.399363947	−0.605730256	OR6N2	2.159198595	0.769880217
ENKUR	1.158178116	−0.731674018	IGKV1-37	−1.590432441	−2.252849712

To better understand the biological processes influenced by BKG, we conducted GO and KEGG enrichment analyses separately for upregulated and downregulated genes. The upregulated aging genes affected by BKG were mainly enriched in biological processes, such as response to interleukin-17L-aspartate transmembrane transport, interleukin-17-mediated signaling pathway, cellular response to interleukin-17, estrogen biosynthetic process, positive regulation of ruffle assembly, negative regulation of cell division, response to corticosterone, and L-glutamate transmembrane transport (Figure 4I). No enriched signaling pathways were identified in the KEGG database. The downregulated aging genes affected by BKG were mainly enriched in processes, such as extracellular matrix organization, extracellular structure organization, cellular response to transforming growth factor beta stimulus, response to transforming growth factor beta, negative regulation of blood coagulation, negative regulation of hemostasis, regulation of nitric oxide-mediated signalz transduction, regulation of chloride transport, negative regulation of coagulation, and collagen fibril organization (Figure 4J). The signaling pathways involved the *PI3K*-*AKT* signaling pathway, ECM-receptor interaction, Rap1 signaling pathway, protein digestion, and absorption (Figure 4K). We also performed gene-gene interaction analysis on these 70 genes and identified COLA1, THBS1, EGF, and PDGFRA as the key genes (Figure 4L). These genes play important roles in the *PI3K*-*AKT* signaling pathway. During aging, the activity of the *PI3K*-*AKT* signaling pathway is enhanced, which may be related to decreased responsiveness to environmental stimuli and changes in cellular function and metabolism. BKG significantly inhibited the expression of COLA1, THBS1, EGF, and PDGFRA, which may be the key mechanism underlying its anti-aging effects.

### The protective effect of BKG on D-gal-induced aging of AC16 cell

**FIGURE 5:**
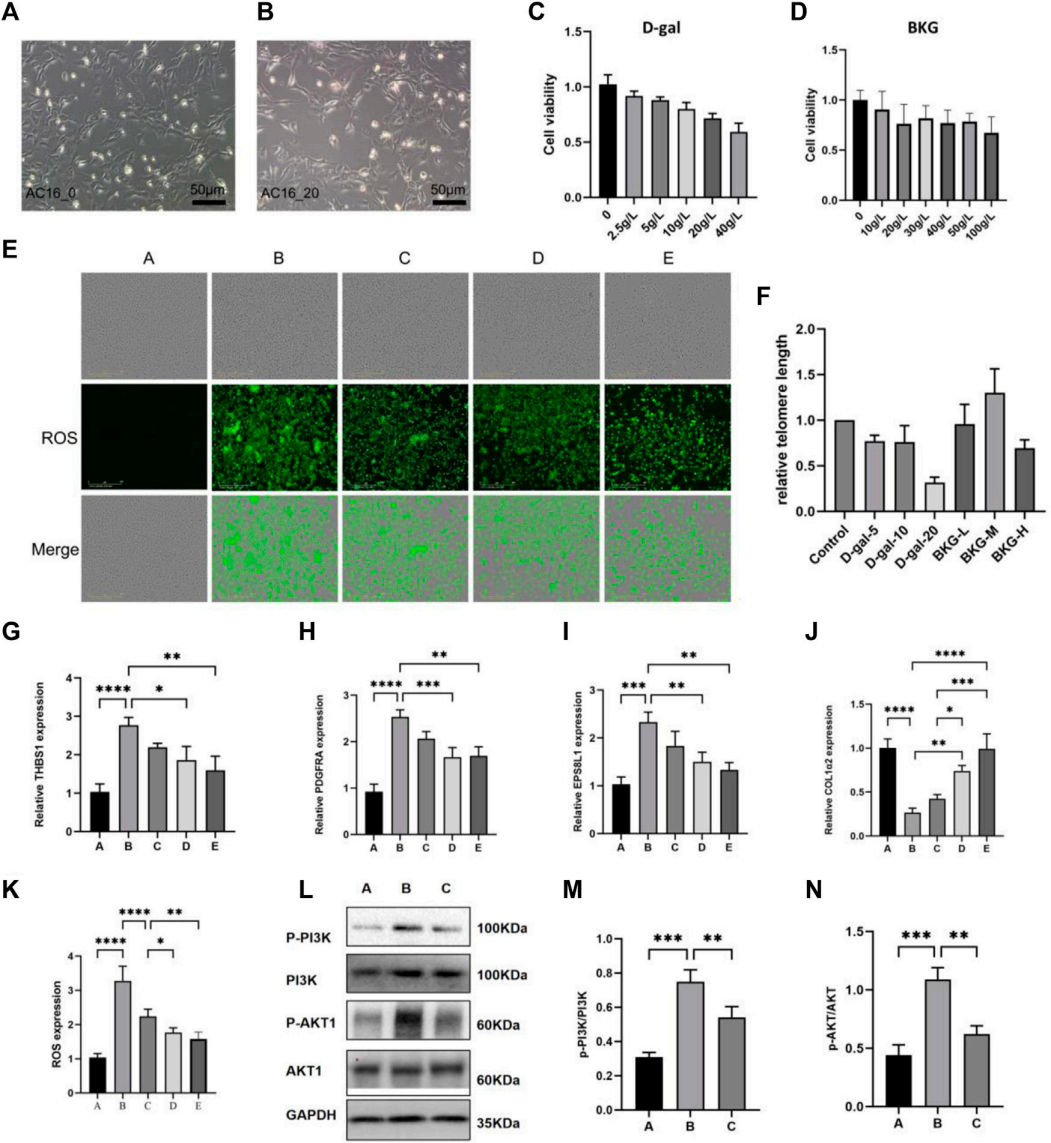
The protective effect of BKG on D-gal-induced aging of AC16 cell. **(A, B)** Shape changes of AC16 cell at 24 h after exposure to D-gal (0 and 20 g/L) were presented as greyscale graphs. **(C, D)** CCK8 experiments were performed at 24 h with different concentration of D-gal (0, 2.5, 5, 10, 20, 40 g/L) and BKG (0, 10, 20, 30, 40, 50, 100 g/L). **(E, K)** The DCFH-DA staining of AC16 cell at 24 h after exposure to D-gal (20 g/L) and BKG (30, 40, 50 g/L). **(F)** RT-qPCR Results of telomere length. **(G–J)** RT-qPCR Results of THBS1, PDGFRA, EPS8L1, COL1A2. **(L–N)** Western blot results of *PI3K*, *AKT*, p-*PI3K*, p-*AKT*. Column graphs are quantification results using ImageJ software. **(A)** Control group. **(B)** Model group. **(C)** BKG (30 g/L) group. **(D)** BKG (40 g/L) group. **(E)** BKG (50 g/L) group.

D-gal is a commonly used drug to induce aging in animals and cells, as reported in previous literature. In order to detect the toxicity of D-gal and BKG on AC16 cells, they were exposed to different concentrations of D-gal (0, 2.5, 5, 10, 20, 40 g/L) and BKG (0, 10, 20, 30, 40, 50, 100 g/L) for 24 h. As showed in Figures 5C, D, cell viability decreased in a dosage-dependent manner in AC16 cells. At dosage of 20 g/L, the viability of AC16 cells decreased to 66%–73% relatively to control. At dosage of 40 g/L, the viability of AC16 cells decreased to 51%–61% relatively to control. In order to ensure that AC16 cells have a certain level of cell viability, we selected 20 g/L as the dosage of D-gal to perform the next experiments. On the other hand, AC16 cells exhibit higher cell viability at BKG concentrations of 30, 40, and 50 g/L. Therefore, we selected 30, 40, and 50 g/L as the low, medium, and high levels of BKG to perform the experiments. To investigate the effect of BKG on ROS production in AC16 cells, we detected ROS expression using immunofluorescence. As shown in Figure 4E; 5K, the expression levels of ROS significantly increased in the model group, while in the BKG groups, ROS expression decreased in a dose-dependent manner. To study the effect of BKG on the aging of AC16 cells, we measured the telomere length of cells using qPCR. As shown in Figure 4F, 20 g/L of D-gal significantly reduced the telomere length of the cells, while the BKG group exhibited a significant increase in telomere length. These results indicate that BKG plays a role in antioxidative stress and in delaying cellular aging. The RT-qPCR was used to detect the impact of BKG on differentially expressed genes. According to the RT-qPCR results (Figures 5G–J), BKG decreased the expression of THBS1, PDGFRA, and EPS8L1 (*p <* 0.05), consistent with the RNA-seq results. Conversely, BKG increased the expression of COL1A2 (*p <* 0.05), contrary to the RNA-seq results. Furthermore, enrichment analysis demonstrated that BKG-related key targets were notably enriched in the *PI3K*-*AKT* signaling pathway. Western blot and quantitative analysis of the *PI3K*-*AKT* signaling pathway in model group revealed a significant increase in the expression levels of p-*PI3K*/*PI3K* and p-*AKT*/*AKT*, compared to control group (*p <* 0.05). Conversely, the BKG group exhibited significantly decreased levels of p-*PI3K*/*PI3K* and p-*AKT*/*AKT*, as compared to the model group (*p <* 0.05), as illustrated in Figures 5L–N. Collectively, these findings provide evidence that the *PI3K*-*AKT* signaling pathway is involved in the therapeutic effects of BKG in a aging model of AC16.

## Discussion

Aging is a physiological process caused by multiple factors that involve multiple systems. It can lead to various age-related diseases, such as cancer, diabetes, osteoporosis, hypertension, and neurodegenerative diseases (la Torre et al., 2023). Recent studies have shown that oxidative stress and chronic inflammation are important characteristics of cellular aging (Liguori et al., 2018; Baechle et al., 2023). With age, mitochondrial function declines and electron leakage increases, leading to an increase in the production of reactive oxygen species (ROS) and a decrease in ATP synthesis (Raha and Robinson, 2000). When the body’s antioxidant repair systems fail to remove excess free radicals, they can damage cell membranes and DNA, inhibit protein function, and increase genomic instability (Fougere et al., 2018). The accumulation of erroneously synthesized DNA in different tissues and cells of the body affects the normal function of cells and tissue repair capability. In addition, cellular and organelle damage releases self-derived fragments known as damage-associated molecular patterns (DAMPs) that activate the innate immune system (Franceschi and Campisi, 2014). Activated immune cells release a large amount of reactive oxygen species, such as superoxide anion (O_2_·-), hydrogen peroxide (H_2_O_2_), and hydroxyl radical (OH) (Nathan and Cunningham-Bussel, 2013). Reactive oxygen species (ROS) have strong oxidizing properties and react with lipids, proteins, and nucleic acids within cells, causing oxidative damage and accelerating aging in tissues and organs. Simultaneously, senescent cells can trigger chronic inflammation. Senescent cells express and secrete various extracellular factors, such as cytokines, chemokines, proteases, and growth factors, known as the senescence-associated secretory phenotype (SASP) (Lopes-Paciencia et al., 2019). The SASP stimulates and recruits immune cells to eliminate potential tumor cells or harmful pathological products, but it can also lead to a chronic state of inflammation, causing disruption of tissue structure and function, disturbance in intercellular communication, and remodeling of the extracellular matrix (Han et al., 2022). In addressing chronic inflammation and oxidative stress processes in aging, some drugs have been developed to slow down the aging process, such as vitamins, glutathione, coenzyme Q10, non-steroidal anti-inflammatory drugs, and cyclosporine A (Duarte and Lunec, 2005; Meydani et al., 2005; Mcgeer et al., 2018; Zhang J. et al., 2019; Liu et al., 2019). However, most of these drugs and compounds are still in the research and experimental stages and their safety and efficacy require further validation. Aging is a complex process involving multiple factors and completely reversing or delaying aging is difficult with a single drug.

Increasing scientific evidence supports the use of TCM in delaying aging (Liu et al., 2020). In this clinical study, on a macroscopic level, the kidney-tonifying and anti-aging formulas significantly improved the symptoms related to aging and fatigue status of the participants. At a microscopic level, the formula increased SOD levels, enhanced antioxidants functioning, reduced TNF-α levels, and alleviated the damage caused by inflammatory reactions in the body. In addition, no significant advantages were observed in the regulation of immune function using this formula. This could be due to the small sample size, which may have affected the accuracy of the clinical results. Moreover, the participants in this study were healthy individuals and their immune levels were within a relatively normal range. Therefore, TCM have a limited effect on the regulation of the immune function. The kidney-tonifying and anti-aging formulas tended to increase hormone secretion, but statistically significant differences were not observed. This could be attributed to the small sample size of this study. Severe adverse events were not observed among the participants during the study period. This formula significantly reduced AST levels after treatment, suggesting a possible hepatoprotective effect. However, the treatment resulted in an increase in Cr levels, which showed statistically significant differences compared to those before treatment. The reason for this remains unclear and may be related to small sample sizes.

**FIGURE 6:**
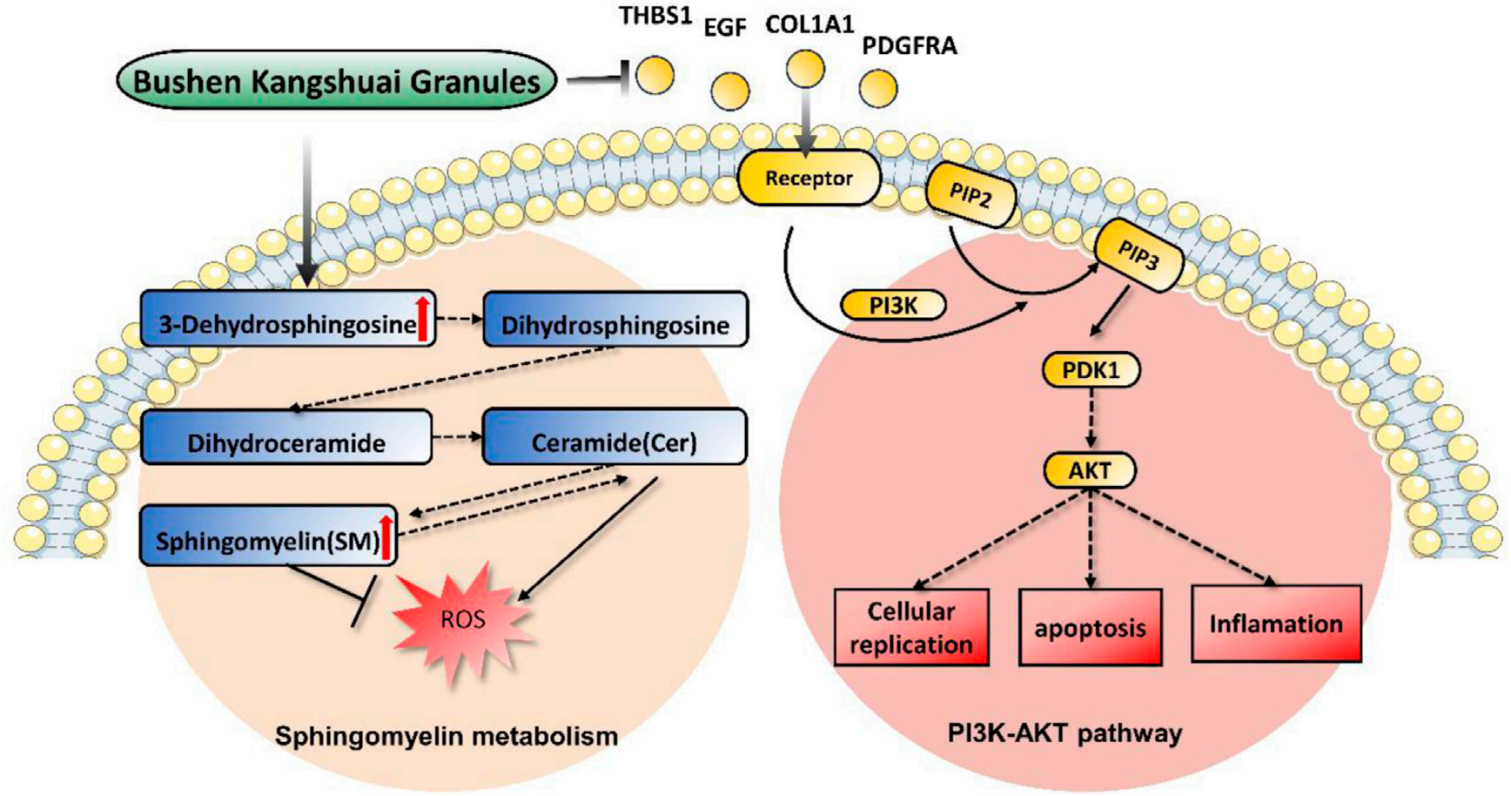
Schematic diagram of the connection between sphingomyelin metabolism and *PI3K* pathway.

Omics technologies have been widely accepted and utilized to study the holistic and dynamic characteristics of TCM and herbal medicine through systematic and integrative approaches (Duan et al., 2018). Our transcriptomic study revealed 6,446 differentially expressed genes between aging and young individuals, and BKG restored the expression levels of 70 genes, including 20 upregulated and 50 downregulated genes. These genes are involved in major pathways, such as the *PI3K*-*AKT* signaling pathway, Rap1 signaling pathway, and choline metabolism in cancer. Overactivation of the *PI3K*/*AKT* pathway promotes cellular replication stress, leading to sustained DNA damage and mitotic abnormalities, and ultimately promoting cancer cell generation. Blocking the *PI3K*/*AKT* pathway can reduce the negative consequences of these effects (Sinha et al., 2020). When mitochondria are unable to repair damage, inhibition of the *PI3K* pathway can activate mitochondrial autophagy, reduce mitochondrial dysfunction, and ensure normal cellular vitality while decreasing ROS production (Onishi et al., 2021). *PI3K* signalling also regulates lipid metabolism. Studies have shown that the crosstalk between the *PI3K* and sphingomyelinase pathways is involved in determining cell survival or death (Burow et al., 2000). The ligands and receptors that activate the *PI3K* pathway include EGF, COL1A1, THBS1, ITGA11, and PDGFRA. In this study, aging patients showed an upregulated expression of COL1A1, EGF, ITGA11, PDGFRA, and THBS1. BKG treatment diminishes the expression of these genes, suggesting that BKG may inhibit the *PI3K*/*AKT* signaling pathway to regulate aging (Figure 6).

Extensive data indicate that lipid metabolism plays a crucial role in aging (Johnson and Stolzing, 2019). Interventions related to lipid metabolism may improve human health and the complex products of lipid metabolism may represent a rich source of biomarkers for human aging. We found significant changes in 3-Dehydrosphingosine, SM(d18:1/22:0), and phosphatidylcholine (PC) levels, which are involved in sphingomyelin (SM), glycerophospholipid, and arachidonic acid metabolism, respectively. SM, the most abundant sphingolipid in cells, is widely distributed in mammalian tissues, with the highest levels found in the central nervous system (Huitema et al., 2004). SM is an important component of the cell membrane that effectively prevents lipid peroxidation (Coliva et al., 2020). Modern research has revealed a close association between SM metabolism, aging, and various age-related diseases, such as Alzheimer’s disease (AD). AD, the most common age-related dementia, is characterized by memory loss and impaired executive function. Studies have shown a general decrease in SM levels in the brain of patients with AD (Söderberg et al., 1992; Gottfries et al., 1996). Through transcriptomic studies, Simone et al. found that genes related to sphingolipid metabolism were downregulated during normal aging and upregulated with the onset of AD (D’Angiolini et al., 2022). In Parkinson’s disease (PD), SM has been identified as an important risk factor for PD development (Sidransky et al., 2009). Furthermore, the prevalence of cardiovascular diseases increases sharply with age, and disrupted sphingolipid metabolism in the heart is considered a prerequisite for age-related cardiac pathogenesis (Geiger et al., 2013; Babenko and Storozhenko, 2017). Elevated levels of mitochondrial ceramides associated with aging lead to decreased cardiac phospholipid content and mitochondrial dysfunction and contribute to the development of myocardial infarction, stroke, and heart failure (Paradies et al., 2010). In the human skin system, SM activity gradually decreases with age, with elderly individuals having only a quarter of the activity of young individuals (Yamamura and Tezuka, 1990). In this study, we found that SM(d18:1/22:0) and 3-Dehydrosphingosine, involved in SM metabolism, were downregulated in the aging population and upregulated after BKG treatment, indicating that BKG improves sphingolipid metabolism and enhances antioxidant function.

Glycerophospholipid metabolism is the synthesis, degradation, and regulation of glycerophospholipids within organisms. Phosphatidylcholine (PC) is a major component of glycerophospholipid metabolism. It is an important component of the cell membrane and plays crucial roles in the maintenance of organ function, lipid metabolism, cell membrane signal transduction, and cell repair. Additionally, it is a target of free-radical attacks (Li and Vance, 2008). Our study found that PC(16:0/22:5 (7Z,10Z,13Z,16Z,19Z)), PC(18:0/22:4 (7Z,10Z,13Z,16Z)), and PC(O-18:1 (9Z)/18:2 (9Z,12Z)) were downregulated in the aging population but were significantly upregulated after treatment. In contrast, PC(20:0/22:6 (4Z,7Z,10Z,13Z,16Z,19Z)) was upregulated in the aging population but significantly downregulated after treatment, indicating that BKG could reverse the altered expression of PC observed in the aging population. Furthermore, our research found that gamma-glutamylalanine was downregulated in aging individuals but was upregulated after treatment. Gamma-glutamylalanine is an intermediate product of glutathione. Glutathione is an important antioxidant in the body that possesses strong resistance to intracellular oxidative stress, neutralizes free radicals, and reduces oxidative damage (Townsend et al., 2003). During aging, the accumulation of free radicals leads to the peroxidation of PC and other polyunsaturated fatty acids, disrupting the membrane structure and depleting glutathione. Our study suggests that BKG can regulate the metabolism of glutathione and phosphatidylcholine, improve the body’s antioxidant system, reduce oxidative stress, and stabilize the cell membrane structure.

### Strengths and limitations

This study aimed to explore the clinical effects and potential mechanisms of action of BKG on aging. This study has several advantages. First, while most aging-related targets were obtained from online databases (e.g., Cellage), this study included both young and aging populations and identified aging-related targets using high-throughput sequencing. These targets were more representative of local ethnic characteristics. Second, by quantifying aging-related genes and metabolites, we determined the trend and magnitude of BKG intervention on these targets, demonstrating its reversing effects on aging-related targets. However, this study has some limitations. First, randomized, double-blind, controlled trials were not conducted to demonstrate the efficacy and safety of this drug. Second, the connections between differentially expressed genes and metabolites are not well established, and an interaction network has not been formed to link the results of the genes and metabolites. Additionally, to further strengthen this study, increasing the sample size and conducting long-term follow-up to assess the long-term safety and benefits of BKG consumption is necessary.

## Conclusion

This study revealed the clinical efficacy of BKG in an intraindividual before-and-after controlled study in an aging population. BKG was able to improve aging-related symptoms, reduce fatigue levels, increase SOD levels, and decrease TNF-α levels. A comprehensive analysis based on transcriptomic and metabolomic studies indicated that BKG could reverse key aging-related genes, and its mechanism may be associated with the inhibition of the *PI3K*-*AKT* signaling pathway. Additionally, after treatment with BKG, the levels of several metabolites that were elevated in aging patients were downregulated. BKG lowered SM levels involved in sphingolipid metabolism and regulated PCs levels involved in glycerophospholipid metabolism. These findings suggest that combining clinical trials with transcriptomics and metabolomics approaches is an effective strategy for evaluating the complex mechanisms of TCM in disease conditions and that the relevant pathway targets require further validation through basic experiments.

## Figures and Tables

**Table:** 

**BKG**	Bushen Kangshuai Granules
**SOD**	Superoxide Dismutase
**TNF-α**	Tumor Necrosis Factor-alpha
**TCM**	Traditional Chinese Medicine
**SAMP8**	Senescence Accelerated Mouse-Prone 8
**ALT**	Alanine aminotransferase
**AST**	Aspartate aminotransferase
**ALP**	alkaline phosphatase
**DIC**	disseminated intravascular coagulation
**BUN**	blood urea nitrogen
**Cr**	Creatinine
**UA**	urine analysis
**EDTA**	Ethylenediaminetetraacetic acid
**PCR**	polymerase chain reaction
**FPKM**	fragment per kilobase of transcript per million mapped reads
**FDR**	false discovery rate
**GO**	Gene Ontology
**KEGG**	Kyoto Encyclopedia of Genes and Genomes
**DEGs**	differentially expressed genes
**QC**	quality control
**ESI**	electrospray ionization
**PCA**	principal component analysis
**OPLS-DA**	orthogonal projection to latent structures-discriminant analysis
**LC-MS**	liquid chromatography–mass spectrometry
**VIP**	value of the projected
**HCA**	Hierarchical cluster analysis
**HMDB**	Human Metabolome Database
**PCA**	Principal component analysis
**ROS**	reactive oxygen species
**DAMPs**	damage-associated molecular patterns
**SASP**	senescence-associated secretory phenotype
**PC**	phosphatidylcholine
**SM**	sphingomyelin
**AD**	Alzheimer’s disease
**PD**	Parkinson’s disease

## Data Availability

The data presented in the study are deposited in the GSA-Human repository, accession number HRA008030. Available at: https://ngdc.cncb.ac.cn/gsa-human/browse/HRA008030.
