# Supplementary material for: Integrating transcriptomics and metabolomics to reveal the protective effect and mechanism of Bushen Kangshuai Granules on the elderly people
Source: Front Pharmacol. 2024 Jul 29;15:1361284. doi: 10.3389/fphar.2024.1361284 (PMC11317404; doi:10.3389/fphar.2024.1361284)
Supplement: Supplementary file 1 [file Table2.docx]

**Integrating transcriptomics and metabolomics to reveal the clinical efficacy and mechanisms of BSAG Granules in treating aging**

Additional file1

Table S1 Aging symptom scale of TCM

|  | |  |  |  |
| --- | --- | --- | --- | --- |
| Number | | First-level indicator | Second-level indicator | Score |
| 1 | | Sore waist and knees | No | □0 |
|  | |  | Mild: Mild waist and knee tenderness, does not affect work and life | □4 |
|  | |  | Moderate: Waist and knee acid and soft heavier, slightly affect the work | □6 |
|  | |  | Severe: The waist and knee are more severe, affecting work and life, and difficult to adhere to | □10 |
| 2 | | Lumbopelvic cold pain | No | □0 |
|  | |  | Mild: Slightly cold waist | □2 |
|  | |  | Moderate: Waist cold obvious, tolerable | □4 |
|  | |  | Severe: Waist cold is unbearable | □6 |
| 3 | | Alopecia | No | □0 |
|  | |  | Mild: Slight hair loss; tooth loosening | □2 |
|  | |  | Moderate: Substantial hair loss; small loss of teeth | □4 |
|  | |  | Severe: Hair thinning; multiple tooth loss | □6 |
| 4 | | Dizziness and tinnitus | No | □0 |
|  | |  | Mild: Occurs occasionally | □3 |
|  | |  | Moderate: Occurs frequently | □5 |
|  | |  | Severe: Occurs frequently and cannot be relieved | □8 |
| 5 | | Night sweats | No | □0 |
|  | |  | Mild: Slightly damp skin | □3 |
|  | |  | Severe: Wet skin or sweaty clothes | □5 |
| 6 | | Dysphoria in chestpalms-soles | No | □0 |
|  | |  | Mild: Slightly damp skin | □3 |
|  | |  | Severe: Wet skin or sweaty clothes | □5 |
| 7 | | Dry eyes | No | □0 |
|  | |  | Mild: Occasionally dry eyes | □3 |
|  | |  | Severe: Often dry eyes | □5 |
| 8 | | Dry mouth | No | □0 |
|  | |  | Mild: Occurs occasionally | □2 |
|  | |  | Severe: Occurs frequently | □4 |
| 9 | | Sensation of chill | No | □0 |
|  | |  | Yes | □1 |
| 10 | | Shortness of breath | No | □0 |
|  | |  | Mild | □1 |
|  | |  | Severe | □2 |
| 11 | | Forgetfulness | No | □0 |
|  | |  | Mild: Occasionally forget things, can still recall | □2 |
|  | |  | Moderate: When see forget things, not easy to remember | □4 |
|  | |  | Heavy: Instant forget, can not recall | □6 |
| 12 | Oedema | No | □0 | |
|  |  | Mild | □1 | |
|  |  | Severe | □2 | |
| 13 | Incomplete emptying after urination | No | □0 | |
|  |  | Mild | □1 | |
|  |  | Severe | □2 | |
| 14 | Enuresis nocturna | No | □0 | |
|  |  | Mild | □1 | |
|  |  | Severe | □2 | |
| 15 | Loose stool | No | □0 | |
|  |  | Mild | □1 | |
|  |  | Severe | □2 | |
| 16 | Sexual dysfunction | No | □0 | |
|  |  | Mild | □1 | |
|  |  | Severe | □2 | |
| 17 | Insomnia | No | □0 | |
|  |  | Mild | □2 | |
|  |  | Severe | □4 | |
| 18 | Red tongue | No | □0 | |
|  |  | Yes | □5 | |
| 19 | Swollen tongue | No | □0 | |
|  |  | Yes | □1 | |
| 20 | Thin pulse | No | □0 | |
|  |  | Yes | □1 | |
| 21 | Deep pulse | No | □0 | |
|  |  | Yes | □1 | |

**
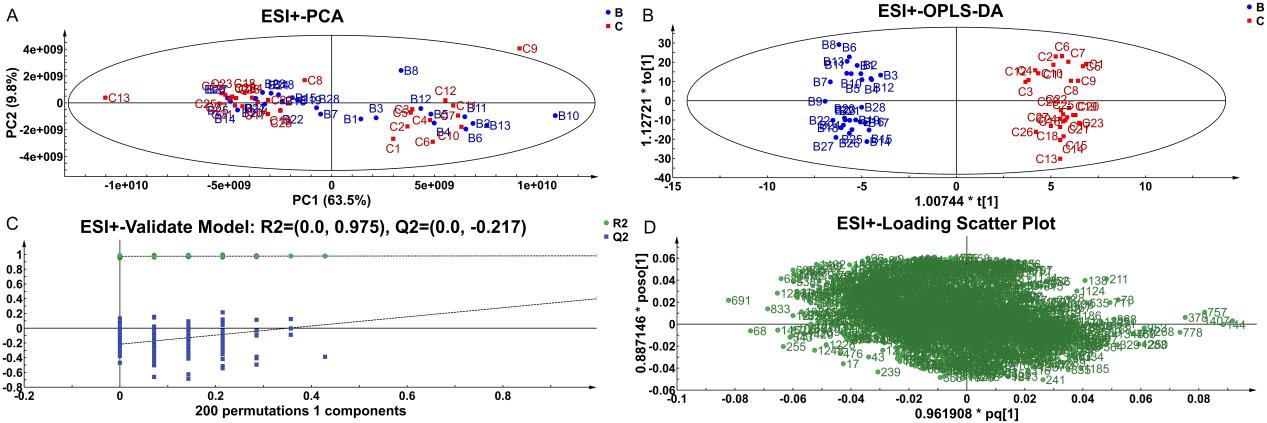
**

**Figure S1.** (A) to (D) were multivariate statistical analysis of B group and C group under ESI+. (A: score scatter plot of PCA under ESI+; B: score scatter plot of OPLS-DA under ESI+; C: Permutation tests under ESI+; D: Loading scatter plot under ESI+).

**Table S2.** Parameters of Multivariate statistic under ESI+

| **Model type** | **Number of principal components** | **Number of orthogonal components** | **Parameter(cum)** | **B vs C** |
| --- | --- | --- | --- | --- |
| PCA-X | 2 | 0 | R2X(cum) | 0.733 |
|  |  |  | Q2(cum) | 0.429 |
| OPLS-DA | 1 | 4 | R2X(cum) | 0.352 |
|  |  |  | R2Y(cum) | 0.981 |
|  |  |  | Q2(cum) | 0.402 |
| Permutation | - | - | R2 | (0.0, 0.975) |
|  |  |  | Q2 | (0.0, -0.217) |


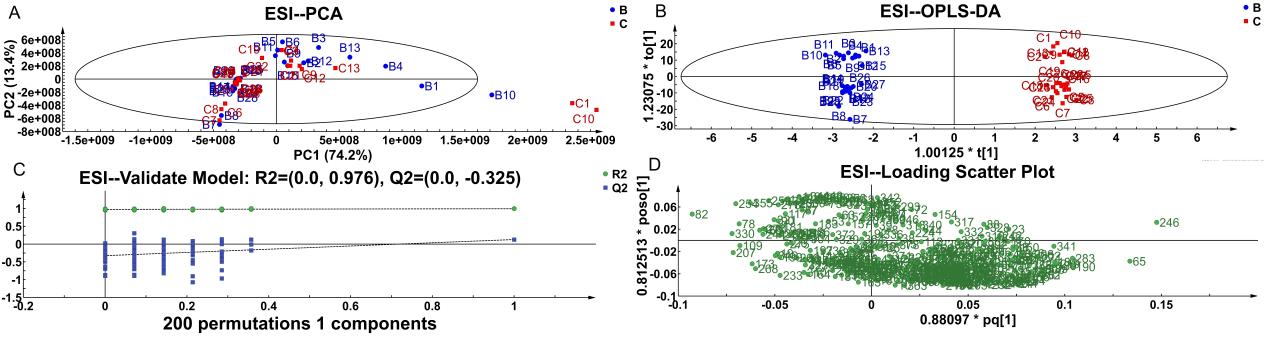


**Figure S2.** (A) to (D) were multivariate statistical analysis of B group and C group under ESI-. (A: score scatter plot of PCA under ESI-; B: score scatter plot of OPLS-DA under ESI-; C: Permutation tests under ESI-; D: Loading scatter plot under ESI-).

**Table S3.** Parameters of Multivariate statistic under ESI-

| **Model type** | **Number of principal components** | **Number of orthogonal components** | **Parameter** | **B vs C** |
| --- | --- | --- | --- | --- |
| PCA-X | 2 | 0 | R2X(cum) | 0.877 |
|  |  |  | Q2(cum) | 0.410 |
| OPLS-DA | 1 | 6 | R2X(cum) | 0.564 |
|  |  |  | R2Y(cum) | 0.994 |
|  |  |  | Q2(cum) | 0.128 |
| Permutation | - | - | R2 | (0.0, 0.976) |
|  |  |  | Q2 | (0.0, -0.325) |

**Table S4.** Differential metabolites between B group and C group (No.1-9:M-H, No.10-79:M+H)

| **No.** | **Metabolites** | **HMDB** | **Formula** | **VIP** | **P-value**  **(C/B)** | **FC**  **(C/B)** | **FC**  **(A/B)** | **Trend**  **(C/B)** | **Trend (A/B)** |
| --- | --- | --- | --- | --- | --- | --- | --- | --- | --- |
| 1 | Testosterone sulfate | HMDB0002725 | C_19_H_28_O_5_S | 1.21 | 0.006 | 7.60 | 5.86 | **↑** | ↑ |
| 2 | N-Formyl aspartate | HMDB0012107 | C_5_H_7_NO_5_ | 2.23 | 0.003 | 2.73 | 4.89 | **↑** | ↑ |
| 3 | EPTC | HMDB0012252 | C_9_H_19_NOS | 1.16 | 0.023 | 0.64 | 3.95 | **↓** | ↑ |
| 4 | Afegostat | HMDB0006248 | C_6_H_13_NO_3_ | 1.25 | 0.027 | 1.89 | 2.02 | **↑** | ↑ |
| 5 | Aceturic acid | HMDB0014903 | C_4_H_7_NO_3_ | 2.43 | 0.001 | 2.34 | 1.20 | **↑** | ↑ |
| 6 | 5-Aminovaleric acid | HMDB0032726 | C_5_H_11_NO_2_ | 1.53 | 0.021 | 1.56 | 1.36 | **↑** | ↑ |
| 7 | 4-Ethyl-2,6-dihydroxyphenyl hydrogen sulfate | HMDB14495 | C_8_H_10_O_6_S | 1.76 | 0.035 | 7.10 | 7.13 | **↑** | ↑ |
| 8 | 1,5-Anhydro-D-fructose | HMDB0254382( HMDB0041931) | C_6_H_10_O_5_ | 1.60 | 0.037 | 1.78 | 1.22 | **↑** | ↑ |
| 9 | (5Z,8Z)-5,8-Tetradecadienoic acid | HMDB0302286 | C_14_H_24_O_2_ | 1.53 | 0.049 | 2.39 | 6.48 | **↑** | ↑ |
| 10 | a-Linolenoyl ethanolamide | HMDB0006469 | C_20_H_35_NO_2_ | 1.42 | 0.020 | 0.81 | 1.12 | **↓** | ↑ |
| 11 | Vitamin A | HMDB0006270 | C_20_H_30_O | 1.80 | 0.011 | 0.78 | 0.88 | **↓** | ↓ |
| 12 | Vigabatrin | HMDB0062656 | C_6_H_11_NO_2_ | 1.77 | 0.002 | 0.61 | 1.16 | **↓** | ↑ |
| 13 | Trans-Cinnamaldehyde | HMDB0253942 | C_9_H_8_O | 1.51 | 0.049 | 0.76 | 0.96 | **↓** | ↓ |
| 14 | TO0127900 | HMDB0000161 | C_9_H_17_NO | 2.18 | 0.009 | 0.50 | 0.51 | **↓** | ↓ |
| 15 | Tetrahydrodeoxycorticosterone | HMDB0002096 | C_21_H_34_O_3_ | 1.74 | 0.043 | 0.79 | 1.23 | **↓** | ↑ |
| 16 | S-Aminoethyl-L-cysteine | HMDB0253066 | C_5_H_12_N_2_O_2_S | 1.93 | 0.010 | 0.75 | 0.90 | **↓** | ↓ |
| 17 | Pyridoxamine | HMDB01492 | C_8_H_12_N_2_O_2_ | 2.60 | 0.0002 | 1.65 | 0.91 | **↑** | ↓ |
| 18 | Pregnanetriol | HMDB0000759 | C_21_H_36_O_3_ | 1.30 | 0.0193 | 0.83 | 0.92 | **↓** | ↓ |
| 19 | PC(O-18:1(9Z)/18:2(9Z,12Z)) | HMDB0033332 | C_44_H_84_NO_7_P | 1.61 | 0.0489 | 1.36 | 1.58 | **↑** | ↑ |
| 20 | O-behenoylcarnitine | HMDB0013785 | C_29_H_57_NO_4_ | 1.98 | 0.0110 | 0.53 | 0.58 | **↓** | ↓ |
| 21 | Norsalsolinol | HMDB0032925 | C_9_H_11_NO_2_ | 1.04 | 0.0091 | 0.80 | 1.01 | **↓** | ↑ |
| 22 | Noradrenaline | HMDB0032610 | C_8_H_11_NO_3_ | 1.97 | 0.0220 | 1.61 | 0.95 | **↑** | ↓ |
| 23 | Naphthalene | HMDB0094660 | C_10_H_8_ | 1.76 | 0.0470 | 0.82 | 1.04 | **↓** | ↑ |
| 24 | Nandrolone | HMDB0032564 | C_18_H_26_O_2_ | 1.56 | 0.0325 | 0.86 | 1.10 | **↓** | ↑ |
| 25 | SM(d18:1/24:1(15Z)) | HMDB0004649 | C_47_H_93_N_2_OP | 1.59 | 0.0211 | 1.73 | 1.44 | **↑** | ↑ |
| 26 | Linoleamide mea | HMDB0033693 | C_20_H_37_NO_2_ | 1.73 | 0.0451 | 0.80 | 0.92 | **↓** | ↓ |
| 27 | gamma-Glutamylalanine | HMDB0031867 | C_8_H_14_N_2_O_5_ | 1.04 | 0.0202 | 1.54 | 1.07 | **↑** | ↑ |
| 28 | Metirosine | HMDB0031770 | C_10_H_13_NO_3_ | 2.21 | 0.0016 | 2.11 | 0.74 | **↑** | ↓ |
| 29 | Menadiol | HMDB0012103 | C_11_H_10_O_2_ | 1.16 | 0.0325 | 0.79 | 0.66 | **↓** | ↓ |
| 30 | Megestrol | HMDB0014382 | C_22_H_30_O_3_ | 1.36 | 0.0339 | 0.85 | 1.07 | **↓** | ↑ |
| 31 | 3,4-Methylenedioxymethamphetamine | HMDB0034437 | C_11_H_15_NO_2_ | 1.35 | 0.0434 | 1.18 | 0.64 | **↑** | ↓ |
| 32 | Malvalic acid | HMDB0033376 | C_18_H_32_O_2_ | 1.65 | 0.0061 | 0.76 | 1.11 | **↓** | ↑ |
| 33 | Linoleyl carnitine | HMDB0032049 | C_25_H_45_NO_4_ | 1.51 | 0.0241 | 0.44 | 0.39 | **↓** | ↓ |
| 34 | Linolenelaidic acid | HMDB0247918 | C_18_H_30_O_2_ | 1.34 | 0.0287 | 0.775 | 1.33 | **↓** | ↑ |
| 35 | Linoleamide | HMDB0059899 | C_18_H_33_NO | 1.27 | 0.0299 | 0.71 | 1.02 | **↓** | ↑ |
| 36 | Lactamide | HMDB0000448 | C_3_H_7_NO_2_ | 1.18 | 0.0489 | 1.09 | 0.69 | **↑** | ↓ |
| 37 | L-(+)-Alanine | HMDB0240773 | C_3_H_7_NO_2_ | 1.86 | 0.0184 | 1.37 | 0.98 | **↑** | ↓ |
| 38 | Indole-3-butyric acid | HMDB0061857 | C_12_H_13_NO_2_ | 1.12 | 0.0416 | 0.60 | 0.67 | **↓** | ↓ |
| 39 | Helenalin | HMDB0040047 | C_15_H_18_O_4_ | 1.63 | 0.0241 | 0.76 | 1.14 | **↓** | ↑ |
| 40 | g-Tokoferol | HMDB0246362 | C_15_H_18_O_4_ | 1.43 | 0.0274 | 0.80 | 1.30 | **↓** | ↑ |
| 41 | Gly-Leu | HMDB0000764 | C_8_H_16_N_2_O_3_ | 1.81 | 0.0078 | 0.78 | 0.87 | **↓** | ↓ |
| 42 | Garcinone D | HMDB0001480 | C_24_H_28_O_7_ | 1.63 | 0.0146 | 0.70 | 1.19 | **↓** | ↑ |
| 43 | Furan | HMDB0013751 | C_4_H_4_O | 1.63 | 0.0286 | 1.37 | 0.69 | **↑** | ↓ |
| 44 | 1-Phenyl-1,3-eicosanedione | HMDB0245294 | C_26_H_42_O_2_ | 1.28 | 0.0416 | 0.79 | 1.25 | **↓** | ↑ |
| 45 | 1-Methyl-4-phenylbenzene | HMDB0035207 | C_13_H_12_ | 1.97 | 0.0100 | 0.76 | 1.01 | **↓** | ↑ |
| 46 | Diethyl phthalate | HMDB0006050 | C_12_H_14_O_4_ | 1.38 | 0.0074 | 0.82 | 0.95 | **↓** | ↓ |
| 47 | Dibenzylmethane | HMDB0032142 | C_15_H_16_ | 2.02 | 0.0052 | 0.77 | 0.95 | **↓** | ↓ |
| 48 | Dehydroretinaldehyde | HMDB0030933 | C_20_H_26_O | 2.43 | 0.0005 | 0.67 | 0.74 | **↓** | ↓ |
| 49 | Dehydrofalcarinone | HMDB0008054 | C_17_H_20_O | 1.53 | 0.0400 | 0.84 | 0.94 | **↓** | ↓ |
| 50 | Celestolide | HMDB0007990 | C_17_H_24_O | 1.53 | 0.0286 | 0.87 | 0.96 | **↓** | ↓ |
| 51 | Carbofuran | HMDB0008288 | C_12_H_15_NO_3_ | 2.41 | 0.0001 | 1.82 | 1.07 | **↑** | ↑ |
| 52 | SM(d18:1/22:0) | HMDB0010395 | C_45_H_91_N_2_OP | 1.71 | 0.0146 | 1.68 | 1.18 | **↑** | ↑ |
| 53 | Butabarbital | HMDB0004667 | C_10_H_16_N_2_O_3_ | 2.19 | 0.0006 | 1.46 | 1.01 | **↑** | ↑ |
| 54 | Biphenyl | HMDB0040806 | C_12_H_10_ | 1.87 | 0.0133 | 0.81 | 0.98 | **↓** | ↓ |
| 55 | Benzyl butyrate | HMDB0011394 | C_11_H_14_O2 | 1.70 | 0.0202 | 0.73 | 0.96 | **↓** | ↓ |
| 56 | Benzophenone | HMDB0032441 | C_13_H_10_O | 1.87 | 0.0161 | 0.76 | 0.96 | **↓** | ↓ |
| 57 | Apocynin | HMDB0002725 | C_9_H_10_O_3_ | 2.01 | 0.0127 | 0.41 | 2.57 | **↓** | ↑ |
| 58 | a-Methylstyrene | HMDB0012107 | C_9_H_10_ | 1.38 | 0.0384 | 0.70 | 1.02 | **↓** | ↑ |
| 59 | Adipic acid | HMDB0012252 | C_6_ H_10_ O_4_ | 1.46 | 0.0168 | 0.71 | 1.02 | **↓** | ↑ |
| 60 | Acetylcarnitine | HMDB0006248 | C_9_H_17_NO_4_ | 1.22 | 0.0312 | 1.43 | 0.47 | **↑** | ↓ |
| 61 | 6-Phenylundecane | HMDB0014903 | C_17_H_28_ | 1.57 | 0.0299 | 0.84 | 1.13 | **↓** | ↑ |
| 62 | 5-Formyl-6-methyl-2,3-dihydro pyrrolizine | HMDB0032726 | C_9_H_11_NO | 1.57 | 0.0312 | 0.76 | 0.94 | **↓** | ↓ |
| 63 | 4-Aminopyridine | HMDB14495 | C_5_H_6_N_2_ | 1.26 | 0.0325 | 1.12 | 1.02 | **↑** | ↑ |
| 64 | 3-Phenylpropanoic acid | HMDB0041931 | C_9_H_10_O_2_ | 1.59 | 0.0384 | 0.76 | 0.96 | **↓** | ↓ |
| 65 | 3-Dehydrosphingosine | HMDB0302286 | C_18_H_35_NO_2_ | 1.72 | 0.0016 | 0.75 | 1.03 | **↓** | ↑ |
| 66 | 2-Pyridone | HMDB0006469 | C_5_H_5_NO | 1.21 | 0.0400 | 0.65 | 0.76 | **↓** | ↓ |
| 67 | 2-Phenylindole | HMDB0006270 | C_14_H_11_N | 1.34 | 0.0416 | 0.61 | 0.95 | **↓** | ↓ |
| 68 | 2-Phenyl-4-pentenal | HMDB0062656 | C_11_H_12_O | 1.92 | 0.0176 | 0.76 | 1.03 | **↓** | ↑ |
| 69 | 2-Hydroxyphenylalanine | HMDB0253942 | C_9_H_11_NO_3_ | 1.18 | 0.0384 | 2.59 | 0.80 | **↑** | ↓ |
| 70 | 2,4-Dimethylbenzaldehyde | HMDB0000161 | C_9_H_10_O | 1.45 | 0.0241 | 0.73 | 0.99 | **↓** | ↓ |
| 71 | 2-(12-Tridecyn-1-yl)furan | HMDB0002096 | C_17_H_26_O | 1.57 | 0.0110 | 0.75 | 1.24 | **↓** | ↑ |
| 72 | PC(18:0/22:4(7Z,10Z,13Z,16Z)) | HMDB0253066 | C_48_H_88_NO_8_P | 1.79 | 0.0071 | 2.19 | 1.66 | **↑** | ↑ |
| 73 | PC(16:0/22:5(7Z,10Z,13Z,16Z,19Z)) | HMDB01492 | C_46_H_82_NO_8_P | 1.88 | 0.0116 | 1.53 | 1.42 | **↑** | ↑ |
| 74 | PC(20:0/22:6(4Z,7Z,10Z,13Z,16Z,19Z)) | HMDB0000759 | C_50_H_88_NO_8_P | 1.87 | 0.0434 | 0.59 | 0.60 | **↓** | ↓ |
| 75 | LysoPC(20:4(5Z,8Z,11Z,14Z)/0:0) | HMDB0033332 | C_28_H_50_NO_7_P | 1.51 | 0.0489 | 0.75 | 0.68 | **↓** | ↓ |
| 76 | 13-HODE | HMDB0013785 | C_18_H_32_O_3_ | 1.46 | 0.0193 | 0.76 | 1.26 | **↓** | ↑ |
| 77 | [7]-Paradol | HMDB0032925 | C_18_H_28_O_3_ | 1.28 | 0.0489 | 0.88 | 1.07 | **↓** | ↑ |
| 78 | PE(P-18:0/22:6(4Z,7Z,10Z,13Z,16Z,19Z)) | HMDB0032610 | C_45_H_78_NO_7_P | 1.63 | 0.0251 | 1.42 | 1.19 | **↑** | ↑ |
| 79 | (2Z,4Z,6Z)- Nonatrienal | HMDB0094660 | C_9_H_12_O | 1.81 | 0.0067 | 0.77 | 0.96 | **↓** | ↓ |

| **Table S5.** Primer list | | Primer sequence**(5'to3')** |
| --- | --- | --- |
| Telomere | Forward primer | ACACTAAGGTTTGGGTTTGGGTTTGGGTTTGGGTTAGTGT |
| Telomere | Reverse primer | TGTTAGGTATCCCTATCCCTATCCCTATCCCTATCCCTAACA |
| HBG | Forward primer | GCTTCTGACACAACTGTGTTCACTAGC |
| HBG | Reverse primer | CACCAACTTCATCCACGTTCACC |
| THBS1 | Forward primer | GCCATCCGCACTAACTACATT |
| THBS1 | Reverse primer | TCCGTTGTGATAGCATAGGGG |
| PDGFRA | Forward primer | TTGAAGGCAGGCACATTTACA |
| PDGFRA | Reverse primer | GCGACAAGGTATAATGGCAGAAT |
| EPS8L1 | Forward primer | CTCCACGTGAAAACGAGCTCTG |
| EPS8L1 | Reverse primer | CGCTGAAGAACTCGGGTCTGTA |
| COL1A2 | Forward primer | TGCCTAGCAACATGCCAATC |
| COL1A2 | Reverse primer | CAGCAAAGTTCCCACCGA |
| GAPDH | Forward primer | CACCCACTCCTCCACCTTTGA |
| GAPDH | Reverse primer | TCTCTCTTCCTCTTGTGCTCTTGC |
